# Assessing the suitable habitat for reintroduction of brown trout (*Salmo trutta forma fario*) in a lowland river: A modeling approach

**DOI:** 10.1002/ece3.4022

**Published:** 2018-04-27

**Authors:** Pieter Boets, Sacha Gobeyn, Alain Dillen, Eddy Poelman, Peter L. M. Goethals

**Affiliations:** ^1^ Department of Applied Ecology and Environmental Biology Ghent University Ghent Belgium; ^2^ Provincial Centre of Environmental Research Ghent Belgium; ^3^ Agentschap voor Natuur en Bos Ghent Belgium

**Keywords:** brown trout, freshwater management, habitat suitability modeling, river restoration, species reintroduction, stream velocity, uncertainty

## Abstract

Huge efforts have been made during the past decades to improve the water quality and to restore the physical habitat of rivers and streams in western Europe. This has led to an improvement in biological water quality and an increase in fish stocks in many countries. However, several rheophilic fish species such as brown trout are still categorized as vulnerable in lowland streams in Flanders (Belgium). In order to support cost‐efficient restoration programs, habitat suitability modeling can be used. In this study, we developed an ensemble of habitat suitability models using metaheuristic algorithms to explore the importance of a large number of environmental variables, including chemical, physical, and hydromorphological characteristics to determine the suitable habitat for reintroduction of brown trout in the Zwalm River basin (Flanders, Belgium), which is included in the Habitats Directive. Mean stream velocity, water temperature, hiding opportunities, and presence of pools or riffles were identified as the most important variables determining the habitat suitability. Brown trout mainly preferred streams with a relatively high mean reach stream velocity (0.2–1 m/s), a low water temperature (7–15°C), and the presence of pools. The ensemble of models indicated that most of the tributaries and headwaters were suitable for the species. *Synthesis and applications*. Our results indicate that this modeling approach can be used to support river management, not only for brown trout but also for other species in similar geographical regions. Specifically for the Zwalm River basin, future restoration of the physical habitat, removal of the remaining migration barriers and the development of suitable spawning grounds could promote the successful restoration of brown trout.

## 1 INTRODUCTION

Ecological water quality reached an absolute minimum status during the 1990s in many European rivers (EEA, 2015; Romero et al., 2016). Both large and small rivers suffered from hydromorphological degradation and were characterized by a poor chemical water quality. Consequently, the diversity and abundance of most aquatic and especially fish species were influenced by these pressures and declined in several west European river basins (e.g., Belpaire et al., 2000; Boets, Lock, & Goethals, 2011; Den Hartog, Van den Brink, & Van der Velde, 1992). Since the enforcement of the European Water Framework Directive (EWFD) in 2000, the ecological water quality has drastically improved in many European waters (EEA, 2015; Hering et al., 2010; Romero et al., 2016), allowing the recolonization and restoration of freshwater biota.

Recently, the status of native freshwater fish species and lampreys in Flanders (northern part of Belgium) was investigated and categorized according to the IUCN Red List Guidelines (Verreycken et al., 2014). The study concluded that five species that were previously categorized as regionally extinct have expanded their area due to the improved water quality and reintroduction programs. Indeed, in Flanders, similar to other countries in Europe, the installation of wastewater treatment plants and the development of river basin management plans have promoted the improvement in water quality, especially in large rivers. However, overall water quality in smaller rivers and streams has improved only marginally since the late 1990s (VMM, 2016), and this, together with a limited restoration of the physical habitat, can possibly explain why several rheophilic species such as river lamprey (*Lampetra fluviatilis* L.), common dace (*Leuciscus leuciscus*), and brown trout (*Salmo trutta* forma *fario*) are still categorized as vulnerable in Flanders.

The EWFD aims to obtain a good ecological status for all surface waters by 2027. For this, further measures are needed (Carrizo et al., 2017; Hering et al., 2010). Currently, the commission on integrated water management, which is responsible for the follow‐up of the water quality in Flanders, has assigned different priorities to river basins. In this way, they want to maximize the effects of the investments and obtain a good ecological status. Those areas categorized as “core areas” are, with some extra efforts, expected to obtain the good ecological status by 2021, whereas “priority areas” are expected to achieve a good ecological status by 2027 (http://www.integraalwaterbeleid.be).

The Zwalm River basin (Central part of Flanders), which belongs to the Upper Scheldt River basin, is designated as a priority area (VMM, 2016). Several headwaters and tributaries of the river basin are included in the Habitats Directive to ensure the protection of rare and endangered species. As a result of restoration efforts (i.e., installation of wastewater treatment plants and the redevelopment of natural banks), both the chemical water quality and physical habitat conditions have improved over the recent years in the middle reaches of the Zwalm River. In addition, the design and (future) installation of several fish passages should make it possible for fish to freely migrate in most stretches of the river and its tributaries, providing possibilities for fish to build up more healthy populations.

In the context of species restoration programs, it is suggested to reintroduce brown trout in the Zwalm River. Brown trout originally occurred in this basin, but largely disappeared since the 1980s due to a decrease in water quality and a loss of migration possibilities. Only a very small relict population remained present in one of the headwaters of the basin (Sassegembeek). Reintroduction was considered since it allows the restoration of a species which is rare in Flanders, it increases biodiversity, and above all the species acts as an “ambassador species” leveraging support for biodiversity conservation.

Currently, no or very basic and limited information (see Dillen, Martens, Baeyens, Van Gils, & Coeck, 2005) is available on the suitability for brown trout in lowland rivers in Flanders. In literature, it is indicated that Brown trout prefers relatively fast‐flowing rivers (average flow velocity of 0.1–0.4 m/s) with a good vegetation cover and an average water depth of 40–60 cm (Armstrong, Kemp, Kennedy, Ladle, & Milner, 2003; Vismara, Azzellino, Bosi, Crosa, & Gentili, 2001), conditions that are present in the Zwalm River basin. Until now, brown trout has only been reintroduced in the Terkleppebeek, a small stream which is part of the Dender River basin, with mixed success (Dillen & Meulebrouck, 2009). Given the good water quality, this small stream was considered potentially suitable for reintroduction. However, no detailed analysis was performed a priori to assess the suitability.

Since resources for river restoration are often limited, it is important to provide clear and robust guidelines and solid research to support decision making. In this respect, habitat suitability modeling proved useful to support decision making in river and conservation management (e.g., Adriaenssens, De Baets, Goethals, & De Pauw, 2004; Guisan et al., 2006; Mouton, Alcaraz‐Hernández, De Baets, Goethals, & Martínez‐Capel, 2011). More specifically, data‐driven and knowledge‐based habitat suitability models have often been used to assess and to predict the area that is suitable for a species to establish and reproduce (e.g., Boets, Pauwels, Lock, & Goethals, 2014; Elith & Leathwick, 2009; Mouton, Schneider, Depestele, Goethals, & De Pauw, 2007). These models are usually developed as a relation between a species and its environment based on occurrence or abundance data and environmental data, but can be complemented with expert knowledge.

The aim of this study was to develop a habitat suitability model to support the decision making for possible reintroduction of brown trout in the Zwalm River basin. To do so, we developed a habitat suitability model based on a three‐step approach going from the development of a conceptual model based on niche and filter theory (1), to model construction with derivatives from a presence data set (2), and finally the search for alternative models with a presence/absence data set and a metaheuristic optimization algorithm (3). This approach was used to obtain flexible, transparent, and performant models with adequate representation of uncertainty. It is important to note that many fields in environmental modeling have discussed the importance of transparent and flexibility model development for management applications (Grimm et al., 2006; Jakeman, Letcher, & Norton, 2006). To our knowledge, this is the first practical application for freshwater fish species.

## 2 METHODOLOGY

**Figure 1:**
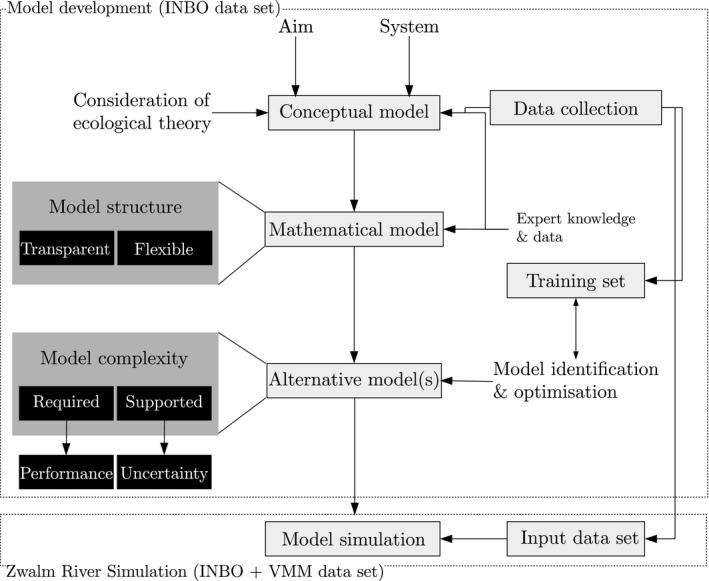
Overview of the methodology used in this paper. The aim of this approach was to develop a model on the scale of Flanders which is applicable for the Zwalm River basin

In Figure 1, the methodology for this case study is illustrated. The aim of this approach was to develop a model for brown trout at the scale of Flanders and apply it for the Zwalm River basin. For this, we considered a conceptual model based on filter and niche theory (see section 2.2) making a minimum set of assumptions on the shape of the species response toward a gradient (i.e., similar as in Maxent (Phillips, Anderson, & Schapire, 2006)). Habitat preference curves (HPCs) were used for the mathematical formulation of the model (see section 2.3). For the estimation of the parameters of these HPCs, the presence data from the Research Institute for Nature and Forest (INBO) and expert knowledge were used (section 2.1). Using and analyzing HPCs as independent model elements, we aimed to increase transparency and flexibility of the model approach (see requirements “Model structure,” Figure 1), facilitating an easy analysis of model elements by stakeholders. Next, a genetic algorithm, a type of metaheuristic algorithm, implemented in the Species Distribution Model Identification Tool (SDMIT) package of Gobeyn, Martin, Dominguez‐Granda, and Goethals (2017) was used to optimize the models with a training data set (see section 2.4). This training data set contained all presence records and an equal number of selected background (i.e., samples where no presence was recorded) records for which observations for all abiotic features were available. The latter was important, since this induced a restriction on the number of available data points for model optimization. By separating the process of mathematical formulation, model construction (section 2.3), and model optimization, we were able to use more data in contrast to when model formulation and optimization would be done in one step. For the Zwalm River basin simulations, an input data set was generated by coupling INBO‐ and Flemish Environment Agency (VMM) data sets. This input data set was used for the simulations for the Zwalm River basin.

### 2.1 Data collection and data processing

**Figure 2:**
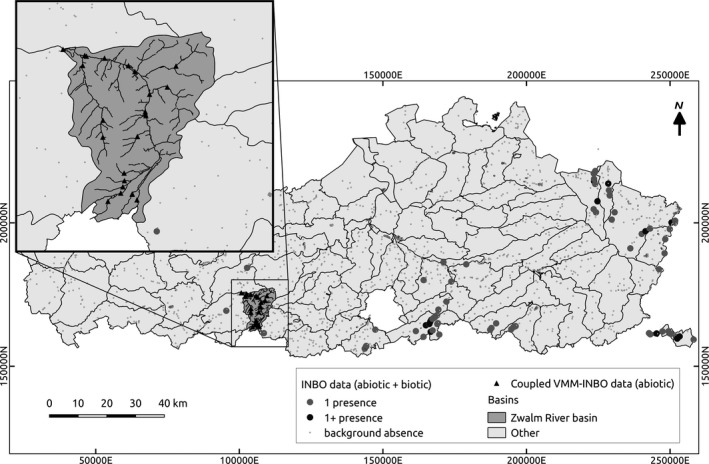
Overview of Flanders and available data to construct and optimize the models (1 presence, 1+ presence, and background absence) and to perform simulations for the Zwalm. The gray and black points indicate the presence of brown trout (1+: more than one observation over time, 1: one observation over time). The small dots indicate background absence data (points where no presence was observed) available. The coordinate system reported on the *x*‐ and *y*‐axis is in Lambert (1972)

For development of the model, we used data that have been collected by the Research Institute for Nature and Forest (INBO) during ongoing monitoring programs to assess the occurrence of fish and to determine the biotic water quality based on fish in Flanders (referred to as “INBO data set”) (Figure 2). Occurrence data of brown trout were retrieved from the Fish Information System (VIS; Brosens et al., 2015) that was accessed from: http://www.gbif.org/dataset/823dc56e-f987-495c-98bf-43318719e30f. Hydromorphological variables linked to the occurrence data were retrieved from INBO as well. For detailed information about the data collection, we refer to Brosens et al. (2015), whereas in supporting information (A), it is explained how data were processed for this case study. Next to data on the occurrence of brown trout, we also used data on the physicochemical water quality of streams and rivers in Flanders. This information was retrieved from the database of the Flemish Environment Agency (VMM) that has been monitoring the water quality in Flanders at more than 2,500 sampling locations since the beginning of the 1990s. Data could be accessed from: http://geoloket.vmm.be/Geoviews/. For a detailed description of the physicochemical data collection, we refer to the Web site of the VMM (https://en.vmm.be/). Physicochemical data were collected eight times a year at different sampling locations resulting in a large number of observations. The data from the VMM were coupled for the Zwalm River basin to the INBO data to increase the number of records used as input for the scenario analysis (section 2.7).

A number of variables were selected for the modeling approach based on five selection criteria: (1) were there sufficient data, (2) was there sufficient variance in the data, (3) was the considered variable ecologically relevant, (4) was the considered variable relevant for management, and/or (5) was the considered variable significantly correlated (5% level) to another variable? An overview of the available data and the processing and explorative analysis steps prior to the variable selection can be found in supporting information (A) and Table 1.

**Table 1:** Overview of the available data and the reason for variable exclusion

Variable	Inclusion	Unit	Values	Reason exclusion
Presence of algae^a^	X	—	Present or absent	
Area		m^2^	Continuous value	Area was related to the sampling area, which was independent of the species presence/absence
Average depth	X	m	Continuous value	
Bank^b^	X	—	Strengthened, partly strengthened, or natural	
River bank slope^b^	X	—	Gradual, average, steep	
Presence of barriers		P/A	Present or absent	Not relevant for management (migration barriers have been removed in the Zwalm, except for which will be solved in the near future)
Brackish		P/A	Yes or no	All considered systems in this study were freshwater systems
Conductivity	X	μS/cm	Continuous value	
Curvature^b^	X		Present or absent	
Dissolved oxygen	X	mg O_2_ L^−1^	Continuous value	
Distance from spring		—	Continuous value	Pooled variables not directly indicating the cause of presence/absence were omitted
Hiding opportunities^b^	X	—	Many, plenty, average, rare or none	
Land use		—	Trees, mixed, agricultural, industry, or city	Only direct pressures were considered
Sampling length		m	Continuous value (usually 100 m)	Length was related to the sampling length, which was independent of the species presence/absence
Presence of nonsubmerged plants^b^	X	P/A	Present or absent	
pH	X	—	Continuous value	
Presence of pools^b^	X	P/A	Present or absent	
Presence of riffles^b^	X	P/A	Present or absent	
Slope of thalweg	X	cm/m	Continuous value	
Presence of submerged plants^b^	X	P/A	Present or absent	
Substrate^b^	X	—	Mixed, fine, sand, stone	
Water temperature	X	°C	Continuous value	
Tidal		P/A	Yes or no	All considered systems in this study were nontidal systems
Transparency		M	Continuous value	Correlation (*r* = .75^c^ with average depth)
Turbidity	X	NTU	Continuous value	
Mean reach velocity	X	m/s	Continuous value	
Water depth		—	?	Insufficient metadata
River width	X	m	Continuous value	
Width of sampling transect		m	Continuous value	Width transect was related to the sampling width, which was independent of the species presence/absence

a Any type of algae (e.g., thread algae) that was visually observed.

b Visual observation/by hand by expert.

c Significant at the 5% level.

From the INBO and VMM data set, three data sets were obtained: one presence data set for model construction of the HPCs (through derivative statistics), one presence/absence data set for model optimization, and one input data set for the simulations. The first data set was compiled from the INBO data set containing all presence instances of Flanders (166 records), whereas in the second set only records which had a value for all abiotic features were retained (25 records). For the latter, 25 records were retained as background samples (thus the training data set had 50 instances). The input data set for the simulations was compiled from coupling the INBO data to the VMM data set (with a delta of 100 m). For the latter, it is important to note that no records of the Zwalm River basin were used for model development.

### 2.2 Conceptual model and theoretical basis

Filter theory and niche theory were used to shape the conceptual model of the suitability models. Using filter theory, we aimed to structure the processes driving species absence in a number of elements (Guisan & Rahbek, 2011; Poff, 1997). Filter theory assumes that the realized species assemblage in a given spatial unit is the result of a number of hierarchical filters, in this study specifically abiotic filters. The concept of niche was used to define each of these abiotic filters in order to reflect the fundamental niche in which species were able to survive. This fundamental niche was assumed to be only shaped by abiotic features and not by dispersal and species interaction effects (Guisan & Rahbek, 2011). In practice, it was highly likely that the realized niche, i.e., niche shaped by species interactions and dispersal, was fitted as this is the niche which was observed in the field. However, Beale and Lennon (2012) stated that it is preferable to model a fundamental niche rather than a realized niche, because the narrower precision of a realized niche likely underestimates model uncertainty. That was why we pursued the idea of reflecting a fundamental niche, so to obtain a more realistic insight into model uncertainty. It is important to note that in this study, we only considered abiotic filtering, as the aim was to assess the habitat preference of brown trout.

### 2.3 Mathematical formulation

Habitat preference curves were used to define the biological response (in this case presence/absence) to abiotic gradients. The abiotic and abundance data of the INBO data set, coupled on location and date, were used to develop these HPCs. It is important to note that abiotic data over the whole of Flanders for which the species was observed as present were used. As explained below in this section, only derivative statistics of the histogram (i.e., percentiles) were used to develop the HPCs. The HPCs described the response of the species over the entire range of abiotic conditions in which the species can survive, so to reflect the fundamental niche. The curves were assumed to have a nonsymmetric unimodal trapezoid shape as a simplification of the bell‐shaped curve. These curves can be asymmetric, allowing to skew away from extreme conditions (i.e., heavily polluted) (Austin, 2007; Guisan & Zimmermann, 2000; Hirzel & Le Lay, 2008). In case of continuous variables, four parameters (*a*_1_, *a*_2_, *a*_3_, *a*_4_) were considered for the HPCs, defining the range and optimal range in which a species can survive. The lower and upper boundaries (*a*_1_ and *a*_4_) were determined by taking the lower and upper values of the environmental variable for which the species was observed. These values were calculated several times to account for uncertainty, by bootstrapping the presence records (i.e., 166 records in total) a number of times (200 bootstraps, until the statistics converged of the bootstraps). The median for the lower and upper values of the bootstraps was taken as the final value for parameters *a*_1_ and *a*_4_. The values for the parameters (*a*_2_ and *a*_3_) defining the optimal range were estimated in a similar manner; the 25 and 75 percentiles of the histogram for which a species was observed as present were calculated. The suitability index (SI) values for a given input variable *x*_*j*_ were then calculated with equation (1), with *j* the index of the variables. It is important to note that the 25 and 75 percentiles were chosen arbitrarily, based on the trade‐off of their robustness to different bootstrap samples (25 percentile will be more robust than 10 percentile) and the optimal range they describe (*x*_*j*_: SI(*x*_*j*_) = 1).

(1) $$\text{SI}\left(x_{j}\right)=\left\{\begin{array}{ccc} 0 & \text{if} & x_{j}<a_{1}\\ \frac{x_{j}-a_{1}}{a_{2}-a_{1}} & \text{if} & x_{j}\in \left[a_{1},a_{2}\right]\\ 1 & \text{if} & x_{j}\in \left[a_{2},a_{3}\right]\\ \frac{a_{4}-x_{j}}{a_{4}-a_{3}} & \text{if} & x_{j}\in \left[a_{3},a_{4}\right]\\ 0 & \text{if} & a_{4}<x_{j} \end{array}\right.$$

For the categorical and binary variables, a SI value per class was assigned by dividing the relative share of the class for which the species was observed as present by the relative share of the class in the data. Afterward, these values were normalized with the maximum obtained value, so an SI value between 0 and 1 was obtained for every class. Also here, bootstrapping was applied on the presence records. The habitat suitability index (HSI) was then defined as the interference of the different HPCs:

(2) $$\text{HSI}={\left(\prod_{j}^{m}\text{SI}\left(x_{j}\right)\right)}^{1/m}$$

where *m* is the number of HPCs considered in the model. The geometric mean was chosen as aggregation function since it was considered to reflect the interference of different environmental factors as defined in the niche theory of Hutchinson (1957). In this model, it was considered that unsuitable conditions (i.e., *x*_*j*_: SI (*x*_*j*_ = 0)), caused by abiotic features, cannot be compensated by other features (Langhans, Reichert, & Schuwirth, 2014). In addition, the multiplied SI values were relaxed by the root (i.e., 1/*m*).

### 2.4 Model optimization and ensemble approach

The SDMIT implemented by Gobeyn et al. (2017) (https://github.com/Sachagobeyn/SDMIT) was used to optimize the habitat suitability model. In this tool, a simple genetic algorithm was used to select the HPCs best explaining the presence/absence of the species. This package was used as it is a flexible, open source package that fits the needs set in Figure 1. The package allows to use a number of objectives and allows a flexible implementation of model structures. In this way, users can define their own model in the code, with assumed distributions, complying with their expert knowledge on species response. For additional information on the algorithm settings and used objective function, we refer to the supporting information (B) and Gobeyn et al. (2017).

For the optimization, the INBO data were used to generate a training data set. Here, background absence samples from the INBO data set were also used, in addition to presence samples used for mathematical formulation (section 2.3). It is important to note that an equal number of presence (i.e., 25) and background records were bootstrap sampled to obtain 50 records and to avoid prevalence dependency of the objective function used for the optimization (Mouton, De Baets, & Goethals, 2010). In addition, the chance that a background record was selected for the training data varied as a function of the distance from the closest presence record. Thus, the farther the background record was situated from a presence record, the lower the chance that a background record was selected. This way, the conditions in case of absence were tested to conditions for presence, in the same geographical unit. As a consequence, the effect of geographical filtering was eliminated.

In order to account for uncertainties caused by the imperfections in the ecological data and the SDMIT analysis, the process of sampling presence/background records and model optimization was repeated a number of times with different sets of the data. This repeated model optimization generated an ensemble of models (Araújo & New, 2007), which was used to reflect simulation uncertainty. The process of optimization was repeated with 200 samples of the data, thus obtaining an ensemble of 200 models (see supporting information [C]).

### 2.5 Model evaluation

Each model was evaluated by calculating a number of evaluation criteria (Table 2) based on the confusion matrix. The confusion matrix is based on binary values, and thus, the HSI values had to be transformed to estimate presence/absence *P*:

(3) $$P=\left\{\begin{array}{ccc} 1 & \text{if} & \text{HSI}\geq \text{threshold}\\ 0 & \text{if} & \text{HSI}<\text{threshold} \end{array}\right.$$

**Table 2:** Used evaluation criteria

Criterion	Symbol	Formula
Cohen's kappa	Kappa	$\frac{\left[\frac{A+D}{N}\right]-\left[\left(A+B\right)\left(A+C\right)+\left(C+D\right)\left(D+B\right)\right]/N^{2}}{1-\left[\left(A+B\right)\left(A+C\right)+\left(C+D\right)\left(D+B\right)\right]/N^{2}}$
Correctly classified instances	CCI	$\frac{\left(A+D\right)}{N}$
Sensitivity	Sn	$\frac{A}{A+C}$
Specificity	Sp	$\frac{D}{B+D}$
True skill statistic	TSS	Sn + Sp−1

*A* is the number of true positives; *B*, the false positives; *C*, the false negatives; and *D*, the true negatives. *N* = *A *+ *B *+ *C *+ *D* (Mouton et al., 2010).

The confusion matrix was used to compute the Cohen's kappa (kappa), correctly classified instances (CCI), sensitivity (Sn), specificity (Sp) and true skill statistic (TSS). The kappa and TSS are statistics that measure interrater agreement for categorical items, normalizing the accuracy of a model by the accuracy that might occur by chance alone, whereas sensitivity and specificity are measuring the share of correct estimation of presence and absence, respectively. In this study, the models were evaluated with the threshold leading to the highest value for the evaluation criteria (TSS). In addition to the described measures, the area under the ROC curve (AUC) was calculated. This measure is often used as a threshold‐independent measure for model performance (Allouche, Tsoar, & Kadmon, 2006; Mouton et al., 2010). The AUC, ranging from 0.5 to 1.0, estimates the discrimination capacity of the model. A model with good discrimination ability is a model that can correctly discriminate between species presence and absence observed in the data. For a model with perfect discrimination, the AUC = 1, and for a model with no discrimination ability, the AUC = 0.5 (Hosmer & Lemeshow, 2000; Pearce & Ferrier, 2000). The models were assessed as well performing for river management when the kappa was higher than 0.6 (Gabriels, Goethals, Dedecker, Lek, & De Pauw, 2007). Based on the AUC, the performance of the models was assessed as poor (AUC ∊ [0.5, 0.7]), reasonable (AUC ∊ [0.7, 0.9]), and very good (AUC > 0.9).

### 2.6 Scenario building

The developed models were used to perform an ensemble simulation of the habitat suitability for brown trout in the Zwalm River basin. The streams in the Zwalm River basin range from nearly pristine headwaters to severely impacted reaches near the mouth of the Zwalm River. Specifically, the physical habitat quality is still excellent in the forested spring areas, but ranges from moderate to poor in the inhabited parts of the river basin due to flood control weirs, straightened river channels, and artificial embankments (Dedecker, Goethals, Gabriels, & De Pauw, 2004).

The input data used for this scenario were based on the data available from the Institute for Nature and Forest (INBO) and the Flemish Environment Agency (VMM) (see section 2.1). First, all INBO and VMM data within the same section of the river were coupled. Data were excluded when the distance between the location of the INBO and the corresponding VMM measurement exceeded 100 m. A distance of 100 m was chosen as this is the resolution at which data were available (i.e., data were collected in the field at reach scale of 100 m). Other thresholds (200, 500 m) were tested; however, it was observed that coverage did not increase. The VMM database was used as a base for the coupling, because the sampling network was denser. Then, a scenario was generated by taking the average state of each variable. In case the median did not coincide with a class (for binary and categorical variables), the worst case scenario was used (e.g., median for algae was 0.5; then, the value 1 (presence of algae) was considered). These compiled data were then used as input for the ensemble models.

## 3 RESULTS

### 3.1 Model development

**Figure 3:**
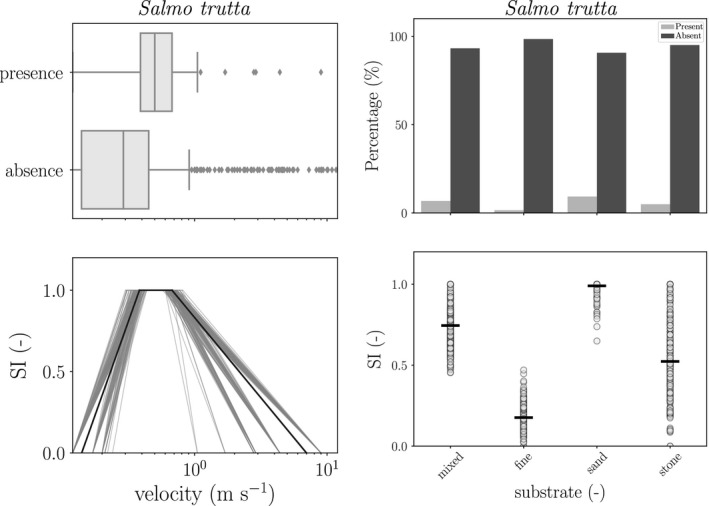
Example of habitat preference curve for a continuous (left, mean stream velocity, m/s) and categorical (right, substrate) variable. In the upper left panel, the boxplot and barplot of the variable values are shown for species presence and absence. In the lower panels, the HPCs derived with the variable values for which the species were present are shown. The different suitability curves were generated by bootstrapping the velocity values of the presence data a number of times. The black curve (line) was determined by taking the median of the *n* values of a_1_, a_2_, a_3,_ and a_4_

In Figure 3, the results of the estimated HPC for mean stream velocity and substrate are shown, whereas in supporting information (A), all plots for the HPCs are shown. For mean reach stream velocity (Figure 3, upper left panel), it is seen that brown trout were—on average—observed at higher velocities, suggesting that their optimal preference for stream velocity was located in rivers with higher velocities (0.2–1 m/s). In addition, there was a larger uncertainty on the upper boundary of the stream velocity of the range than on the lower boundary. For substrate (Figure 3, right lower panel), the classes sand, stone, and mixed had a relatively high SI, whereas for fine sediments (clay and silt), the SI was rather low, since no observations for these classes were available. In addition, a large uncertainty was observed on the SI values of the classes mixed and stone.

**Figure 4:**
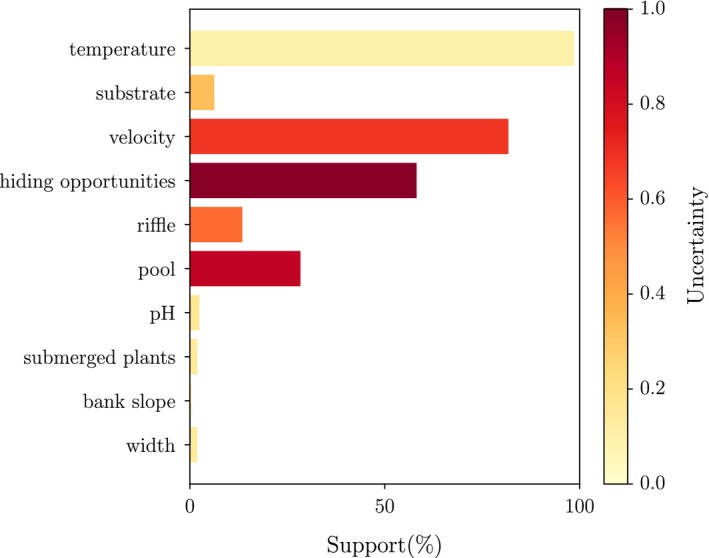
Support and uncertainty on support (Shannon entropy) of HPCs, analyzed for 200 models. The support (in %) is given on the *x*‐axis, while the uncertainty on input variable selection is shown by the color scale (yellow to red, see color print). The Shannon entropy was selected as measure for uncertainty

The support and uncertainty for the HPCs identified with the SDMIT package, analyzed for 200 models, are shown in Figure 4. Water temperature, mean stream velocity, hiding opportunities, presence of pools and to a small extent presence of riffles were identified as steering factors determining the presence or absence of the species. The variable water temperature had the highest support (almost 100%), whereas the variable stream velocity had the second highest support. Hiding opportunities and the presence of pools were identified as the third and fourth important explanatory variable. Although they were assessed as explanatory features steering the presence, the uncertainty on the selection of the input variable “presence of pools” and specifically “hiding opportunities” was assessed as rather high. This uncertainty is reflected in the Shannon entropy (Shannon, 1948), indicating that the inclusion in 50% of the models leads to an uncertainty of one (on the scale of zero to one). The source of this uncertainty stems from the stochastic behavior of the simple genetic algorithm used in SDMIT and the uncertainty introduced by bootstrapping data. The obtained uncertainties can be assessed as high, especially for factors that tend to have some explanatory power. This shows the importance of using the ensemble approach, reflecting uncertainties in species presence estimates. Other factors were excluded from the model, with a higher certainty.

**Figure 5:**
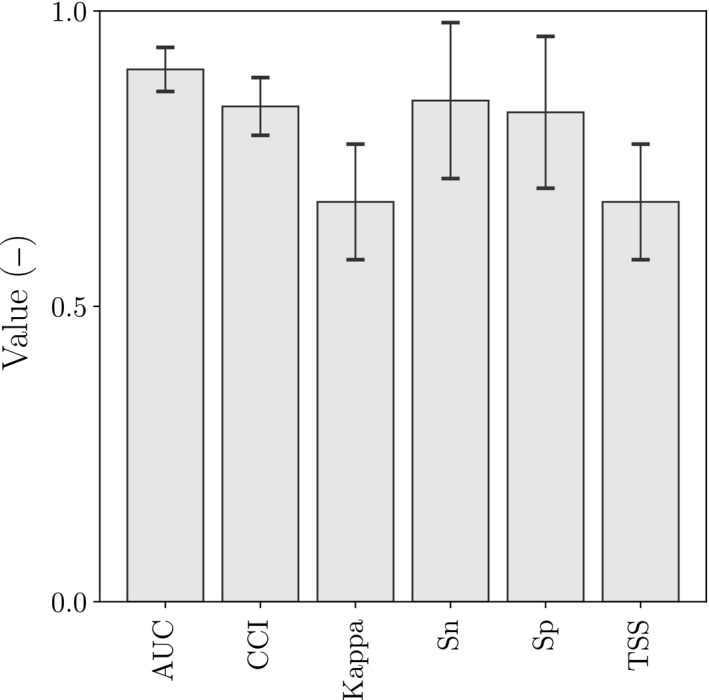
Values for the evaluation measures. The uncertainty on the evaluation measures is given by the standard deviation on the measure (for the abbreviation of the evaluation measures, see Table 2)

An overview of the accuracy of the 200 models is given in Figure 5. The accuracy was evaluated with the threshold that led to the highest TSS (on average this threshold was equal to 0.6). The uncertainty on the values is given by the standard deviation. Presences were fitted better than the absences (i.e., $\bar{\text{Sn}}>\bar{\text{Sp}}$); however, the standard deviation on the values shows that this difference was characterized by uncertainty. The ensemble of models was assessed as performant giving equal weight to the estimation of species presence and absence. Based on kappa, the performance of the models was assessed as moderate and substantial (∊ [0.4, 0.8]), whereas for AUC reasonable (∊ [0.7, 0.9]) and very good (>0.9).

### 3.2 Ensemble forecast for the Zwalm River basin

**Figure 6:**
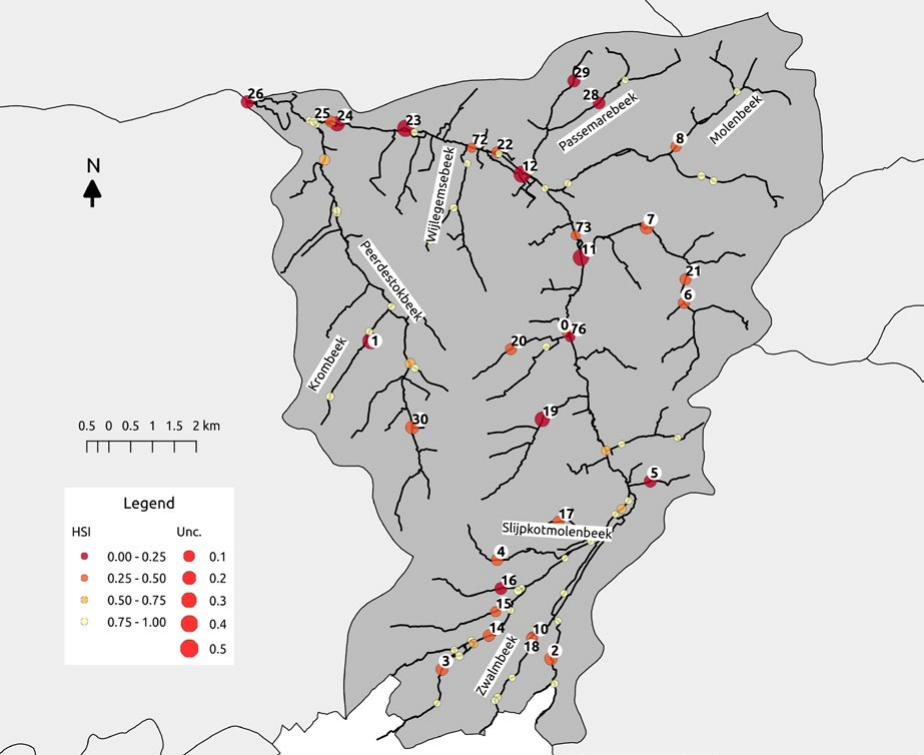
Overview map of simulated HSI with the ensemble of models. In this map, the mean simulated HSI (color red to yellow) over the 200 ensemble models is shown. The uncertainty (Unc.) on the simulation is estimated by computing the standard deviation of the HSI over the 200 simulations (indicated by the size of the points). The ID of the point is indicated when the HSI of the point was lower than 0.6

The model ensemble was used to run a simulation of the habitat suitability of the Zwalm River basin for brown trout. In order to do so, a scenario for the Zwalm was created (see section 2.6). In Figure 6, a map shows the mean of the simulated HSI values and the uncertainty for a number of points in the Zwalm River basin. In Table 3, the results of the minimum of ensemble simulated SI values for every input variable are shown. In this table, only points with a HSI lower than 0.6 are reported, since this was the median threshold which maximizes the evaluation criteria (TSS, see section 2.6).

**Table 3:** Overview of mean habitat suitability index (HSI) values and minimum of suitability index values per variable over 200 ensemble models, this for 30 selected points, based on the mean HSI (lower than 0.6, see also Figure 6)

Point	*X*	*Y*	HSI	Uncertainty	River width	Bank slope	Nonsubmerged plants	pH	Pool	Riffle	Hiding opportunities	Velocity	Substrate	Temperature
1	103,218	170,627	0	0.39	0.99	0.4		0.97				0		1
5	109,025	167,662	0	0.14	0			1						1
11	107,616	172,322	0	0.34	0.97			1			0.7	0	0.74	1
12	106,395	174,067	0	0.39	1	0.4		1				0	0.17	1
16	105,880	165,450	0	0.13	0	0.58		1	1	1	0.7	0.48	0.74	1
19	106,778	168,964	0	0.27	0	0.4		1	1	1	0.25	0	0,74	1
23	103,975	175,038	0	0.38	0.89	0,58		1	1	1	0.99	0	0,17	1
29	107,497	175,999	0	0.14	0			1						1
24	102,551	175,145	0.1	0.26	0.97	0.4		1				0.1		1
28	108,015	175,532	0.17	0.11	0.03			1						1
26	100,685	175,621	0.25	0.17	1	0.58	0.54	1	0.35	0.37	0.25		0.74	1
76	107,374	170,669	0.3	0.04				1						0.3
6	109,757	171,364	0.33	0.09	0.11			0.97						1
17	107,094	166,823	0.33	0.09	0.11			1						1
20	106,132	170,427	0.33	0.09	0.11			1						1
21	109,793	171,856	0.33	0.09	0.11									1
2	106,918	163,978	0.41	0.15	0.11	0.58	1	1	1	1	0.99	0.27	0.99	1
3	104,639	163,781	0.42	0.13	0.09	0.58	0.54	1	1	1	0.99	0.44	0.17	1
25	102,410	175,197	0.42	0.09	1	0.58	1	1	0.35	0.37	0.7	0.56	0.74	1
7	108,992	172,937	0.44	0.19	0.34	0.58	1	0.95	1	1	0.25	1	0.74	1
30	104,060	168,817	0.44	0.19	0.34	0.58		1	1	1	0.25	1	0.74	1
22	105,879	174,517	0.44	0.09	1	0.58	1	1	0.35	0.37	0.7	0.65	0.74	1
0	107,344	170,750	0.45	0.12	1	0.58	0.54	1	1	0.37	0.7	0.29	0.54	1
18	106,514	164,403	0.45	0.06	0.21			1				1		1
72	105,357	174,623	0.46	0.03				1						0.46
4	105,809	166,048	0.48	0.1	0.19	0.58	0.54	1	1	1	0.7	0.42	0.74	1
15	105,770	164,967	0.49	0.06	0.24			1				0.94		1
14	105,623	164,466	0.53	0.1	0.21	0.58	0.54	1	1	1	0.7	0.86	0.17	1
73	107,506	172,785	0.55	0.03				1						0.55
8	109,614	174,613	0.57	0.07	0.26	0.58	0.54	1	1	1	0.7	0.88	0.74	1

Minimum SI values under the value of 0.25 for variables with a support higher than 50% are indicated in gray. No SI values reported for the variables indicate that no input data were available.

A relatively large number of locations were assessed as suitable for brown trout. When using a suitability threshold of 0.6, brown trout was estimated to be present in 48 locations, whereas absent in 31 locations. For a scenario favoring overestimation (HSI > 0.2) and underestimation (HSI > 0.8), the species was estimated to be present in respectively 69 and 42 locations. Suitable locations were mainly concentrated in the side branches (tributaries) and not in the main river (i.e., northeast, Molenbeek, in the south, upstream in the Slijpkotmolenbeek, Zwalmbeek, and Wijlegemsebeek or in the southwest in Paardestokbeek and Krombeek). In general, a large number of unsuitable locations were estimated to be present in the main river. Interestingly, a number of unsuitable locations were simulated to be located upstream in the River basin (i.e., point 3, 14, 15, 4, 10, 18, 2). When inspecting these locations (Table 3), stream velocity, substrate, and river width were identified as limiting variables. For all these points, the stream velocity was in general suboptimal, being too low (∊ [0.21–0.39] m/s); for points 3 and 14, the substrate was fine, which was nonoptimal (Figure 3). In the main river, the habitat of points 0, 76, 11, 12, and 22–26 was less suitable for brown trout, with absence of pools/riffles, fine substrate, and a low mean stream velocity leading to unsuitable conditions. As for the upstream tributaries (south part of the River basin), the low stream velocity was causing these suboptimal conditions. In addition, for points 22 and 25, the absence of pools/riffles was assessed as an important factor influencing the suitability of the habitat. Fine substrate (points 12 and 23) was also assessed as nonoptimal. It was observed that the conditions for temperature were only suboptimal in three of the 31 investigated points (Table 3; locations 72, 74 and 76), ranging from an average 15.85–17.85°C (see supporting information [A], Figure A9). It is important to note that the uncertainty for points with a low HSI was generally higher. Typically, these conditions refer to conditions on the slope of the suitability curves (see Figure 3), which were in general more uncertain than the optimal ranges (i.e., *x*: SI(*x*) = 1).

## 4 DISCUSSION

### 4.1 Model development

In this study, we developed data‐driven habitat suitability models to assess the locations that are suitable for reintroduction of brown trout in the Zwalm River basin. The aim was to provide performant models in terms of precision and accuracy, but also flexibility and transparency of the model structure. We followed a three‐step approach: (1) the development of a conceptual model based on niche theory and filter theory to model construction with derivatives from a presence data set, (2) the search for alternative models with a presence/absence data set, and (3) the use of an optimization algorithm. Although the final end‐product was mainly data driven, the model does reflect prior knowledge embedded in niche and filter theory in its model concept. In contrast to many available approaches used to model species’ occurrence and thus also brown trout (Filipe et al. 2013; Mostafavi et al., 2014), we explicitly split the model construction and optimization phase, to increase model flexibility and transparency. This allows a critical review in every stage of model development (Grimm et al., 2006; Jakeman et al., 2006). It is expected that fully reporting the model development process is beneficial for model developers and those relying on model‐based insight and model recommendations to make decisions. In this way, the proposed approach complements to this idea, in the context of habitat suitability modeling, and it is the first to present a practical application for reintroduction of a freshwater species.

Data‐driven models have been previously shown to be very useful in predicting the habitat preference of fish (Ahmadi‐Nedushan et al., 2006; Mouton et al., 2011; Muñoz‐Mas, Martínez‐Capel, Schneider, & Mouton, 2012). In our study, the boundaries of the HPCs were based on the median of the minimum and maximum values of the environmental field data, whereas the optimal range was based on the 25 and 75 percentiles of these data (i.e., derivative statistics). The four different parameters of the HPC were determined purely data driven, but the choice to use the 25–75 percentile values as the optimal range for brown trout was based on knowledge. Although a similar approach to determine the habitat range was used in previous studies, several studies used the 95% confidence level to set tolerance limits (e.g., Strakosh, Neumann, & Jacobson, 2003). We believe that by using the 25–75 percentile approach we get an optimal range that is biologically more relevant and closer to reality compared to the 95% confidence limit. Recent research by Muñoz‐Mas et al. (2012) showed that the preference intervals of brown trout based on data‐driven HPCs are rather restricted, compared to other studies. Therefore, they also suggest to apply some expert knowledge to set the optimal occurrence range, especially when the data are scarce and when reliable information or scientific experience is available in other formats. The outer range of the suitability was defined by the bootstrapped minimum and maximum value of the abiotic variables. The minimum and maximum values were selected in order to approximate the fundamental rather than the realized species niche. This led to higher observed uncertainties, but is considered more realistic from an ecological point of view (Beale & Lennon, 2012).

To develop the final habitat suitability model, the interference of the different HPCs was used. Afterward, a simple genetic algorithm implemented in the SDMIT package of Gobeyn et al. (2017) was used to optimize our model and to identify alternative models by reducing the number of input variables and decreasing model complexity and risk of overfitting (see also D'heygere, Goethals, & De Pauw, 2006; Gobeyn et al., 2017). In addition, appropriate selection of input variables not only is important for modeling objectives as such, but also ensures reliable decision support in river management and policy‐making (D'heygere et al., 2006). Although water temperature, mean stream velocity, hiding opportunities and presence of pools were identified as the most important variables explaining the occurrence of brown trout, they had a relatively high uncertainty. Therefore, it is suggested to apply an ensemble approach, reflecting uncertainties in species predictions (Araújo & New, 2007; Muñoz‐Mas, Martínez‐Capel, Alcaraz‐Hernández, & Mouton, 2017), as used in this study. The performance of the ensemble models generated in this study could be considered good since the average performance of the different performance criteria was higher than 0.7. Indeed, previous studies have shown that CCI values higher than 70% and kappa values higher than 0.6 indicate reliable models (Gabriels et al., 2007; Mouton et al., 2010).

The ensemble of models was finally used to run a simulation of the habitat suitability of the Zwalm River basin for brown trout. Because outcomes of habitat suitability may have significant consequences for management and reintroduction of species, it is crucial to have insight into the uncertainties of the estimations. The results indicated that the uncertainty for locations with a low HSI is generally higher, whereas for locations with a high HSI the uncertainty is lower. This indicates that the conditions where brown trout does not occur are less clear and probably are characterized by a wider environmental range compared to the conditions that are favorable for brown trout to occur. When the input data lie on the steep parts of the curves, input uncertainty largely determines the uncertainty of the HSI (Van der Lee, Van der Molen, Van den Boogaard, & Van der Klis, 2006). At these positions on the curves, small deviations in input data cause a large variation in the resulting HSI. Nevertheless, the reliability of the HSI obtained in this and other studies is often sufficient for management purposes since the aim is to generally assess potential locations for rehabilitation or conservation activities (Van der Lee et al., 2006). In this regard, our final model could be considered acceptable and fit for purpose.

The developed model serves as an indication for suitability, as no validation data were available to verify its robustness. Future sampling campaigns in the Zwalm River basin are planned, and at that stage, the value of this model can be properly evaluated. Not only can this verify its use for the Zwalm River basin, but also its value for the whole of Flanders. Even more, the modeling approach could serve as a guide to develop models allowing interaction with stakeholders. This way, suggestions and improvements formulated, allows the models to be supported by a larger audience (i.e., policy‐makers).

### 4.2 Habitat suitability variables

Although the habitat suitability of brown trout has been studied before, most studies only considered hydromorphological river characteristics (e.g., Strakosh et al., 2003; Vismara et al., 2001) to assess the optimal occurrence conditions for brown trout. In this study, hydromorphological, chemical, and physical variables were included in the analysis to investigate the habitat suitability for reintroduction of brown trout in the Zwalm River basin. Water temperature, mean stream velocity, hiding opportunities, and presence of pools were selected by the model as the most important variables explaining the occurrence of brown trout. Previous research on the habitat suitability of brown trout in the southern parts of Europe and in the USA indicated that water temperature and stream velocity, two key variables selected by our model, are indeed important variables determining the occurrence and abundance of the species (Mouton et al., 2011; Muñoz‐Mas, Vezza, Alcaraz‐Hernández, & Martínez‐Capel, 2016; Strakosh et al., 2003; Vismara et al., 2001).

Recent research has indicated that water temperature and a species thermal niche are considered important factors determining the maximum distribution of brown trout in Spanish Mediterranean rivers (Santiago et al., 2015). In the central part of the Iberian Peninsula, it was found that the thermal niche of the species is set at a maximum of 18.7°C, whereas its physiological maximum is set at 25°C (Santiago et al., 2015). In our study, the optimal water temperature that is preferred by brown trout is situated between 7 and 15°C, whereas the maximum temperature at which the species still occurred is situated around 20°C. Although our model indicated temperatures between 15 and 17°C as suboptimal, these values are still below the thermal niche of adult brown trout in Spanish Mediterranean rivers. However, research has indicated that with increasing temperatures the species requires higher oxygen concentrations (Raleigh, Zuckerman, & Nelson, 1986), which could explain why temperatures between 15 and 17°C were indicated as suboptimal. The watercourses in the Zwalm River basin are typically small fast‐flowing streams and rivers with a relatively high vegetation cover. The upper parts of the Zwalm River basin are fed by sources which supply groundwater at a steady cool temperature the whole year round. These characteristics explain why the water temperature is relatively low, even in summer when air temperatures rise and water temperatures are still well below the maximum.

In our study, the optimal velocity ranged between 0.2 and 1 m/s, whereas other studies indicated that the optimal range for brown trout is situated lower, with a maximum of around 0.2 m/s (Strakosh et al., 2003; Vismara et al., 2001). Vismara et al. (2001) found that especially juveniles prefer lower stream velocities, but that adults also occurred at microhabitats which are relatively deep and have a high stream velocity. In contrast, Ayllón, Almodóvar, Nicola, and Elvira (2009) found that much depends on the local habitat conditions and the life stage since older trout prefer slower and deeper waters, whereas young‐of‐the‐year showed a strong preference for shallow habitats with a higher stream velocity.

Besides stream velocity and water temperature, the presence of pools and hiding opportunities seemed to be two important factors which were included in some of our models. Previous research has indicated that the presence of pools and hiding opportunities becomes more important (compared to stream velocity) as trout grows and becomes older (Ayllón et al., 2009). Ayllón et al. (2009) found that the interaction between presence of pools and stream velocity seems to be driven by the structural overhead cover and the type of water (fast vs. slow running waters). In addition, the influence of cover on habitat selection remains along the whole life cycle of brown trout, being probably the most important single‐site attribute determining salmonid abundance (Armstrong et al., 2003). Although reproduction was not considered in this study, brown trout needs gravel beds to spawn (Louhi, Mäki‐Petäys, & Erkinaro, 2008). In this respect, the presence of riffles is very important not only as suitable habitat for juveniles and adults, but also since it serves as a habitat for spawning.

In this study, we considered to assess the preference of juveniles complementary to pooled adult/juvenile models to obtain an insight into the essential components driving a juvenile population toward a stable multigeneration population. As no information was available in the data set on the age of the population, a length of 15 cm (based on De Laak, 2008) was used to differentiate juveniles from adults leading to 31 presence records useful for model construction (in contrast to 166 when using all samples). With these samples, HPCs were constructed, showing minor deviance from the HPCs developed using all presence records (see supporting information [D]). In general, the range of the HPCs was smaller and uncertainties were lower. In addition, absence of pools and riffles and a preference for lower stream velocities (see supporting information [A]) were observed for juveniles. We aimed to optimize the models as we did with all presence data from INBO; however, there were not enough records with a value for all abiotic variables. This leads to a training set holding six records which was considered as inadequate for training. As a consequence, no difference was made between juveniles and adults and the simulations were based on a combination of both, which might explain why our results are somewhat different in comparison with previous research.

The assessment of longitudinal connectivity of the river system and the effect of dams and weirs is an important aspect for restoration and conservation of freshwater fish species (Branco, Segurado, Santos, & Ferreira, 2014). Migration speed of the species or the presence of physical migration barriers was not considered in the current study. First of all, several investments have been made during the last decades in order to install bypasses or fish ladders to overcome physical migration barriers which were present in the Zwalm River basin. Currently, only a few bottlenecks remain present mainly on the tributaries and only one migration barrier is still present on the main stem of the River Zwalm (for an overview of the barriers refer to: http://vismigratie.vmm.be/vismigratie/). These migration barriers have been inventoried, and plans have been made to remediate these in the next 5–10 years. Once this handful of migration barriers (mainly present on the tributaries) is resolved, fish will be able to migrate freely in the entire Zwalm River basin and even migration from the river Scheldt will be possible. Second, migration speed was not considered since previous research conducted by Ovidio, Baras, Goffaux, Birtles, and Philippart (1998) in the southern part of Belgium has indicated that brown trout can migrate up to 5 km per night. Given the relatively limited size of the Zwalm River basin (11,650 ha) and the limited length of the Zwalm River (22 km), migration was not considered a limiting factor for the species to reach all suitable habitats within the River Zwalm.

### 4.3 Suitable habitat for reintroduction and recommendations for future management

Based on the ensemble model simulations, we found that mainly the headwaters and some of the tributaries of the Zwalm River basin are suitable for reintroduction of brown trout, whereas the main river is less suitable. The major limiting factor for the main river seems to be stream velocity, which is often too low. The locations and by extension several stretches of the Zwalm River basin indicated as suitable are in agreement with expert knowledge and information retrieved from earlier studies. The tributaries and upper reaches of the Zwalm River basin are characterized by a good physical habitat and a good chemical and ecological water quality (Dedecker et al., 2004; VMM, 2016). Earlier introductions of other rheophilic species seem to thrive well in the headwaters as well (Van Thuyne, Samsoen, & Breine, 2005). Moreover, these small streams are abundantly populated with amphipods and other macroinvertebrates as well as prey fish which could serve as food for brown trout.

In contrast to the physical requirements, which still cause a limitation for brown trout to occur in the Zwalm River basin, the standard chemical water quality conditions (pH, conductivity, dissolved oxygen) were estimated not to be a major restriction for the species to occur. Recent investments in wastewater treatment installations in combination with hydromorphological restoration programs seem to have a positive effect on the suitable habitat, not only of brown trout, but also of other rheophilic species such as dace and chub. Indeed, recent investigations have shown that the reintroduction of both species seems to be successful in the Zwalm River basin (Dillen & Vlietinck, 2008; Van den Neucker et al., 2013). The major challenge remains reproduction and getting a sustainable population, as the spawning grounds are still limited. Therefore, future investments and water management programs should not only focus on an improvement in the habitat for adults and juveniles, but also on the restoration of available spawning grounds.

In conclusion, habitat suitability modeling can be used as an important tool to support the reintroduction of species. Our results indicate that several locations within the Zwalm River basin are suitable for the reintroduction of brown trout. Water temperature and stream velocity are the most important variables determining the habitat suitability for brown trout in Flanders. Future management should focus on the remaining migration barriers mainly present in the tributaries, the improvement in the hydromorphology, and especially the development of suitable spawning grounds.

## CONFLICT OF INTEREST

We have no conflict of interest to declare

## AUTHORS’ CONTRIBUTIONS

Pieter Boets, Eddy Poelman, Alain Dillen, and Peter L.M. Goethals conceived the idea and scope of this paper. Sacha Gobeyn and Pieter Boets designed the methodology and analyzed the results. Pieter Boets collected the data. Pieter Boets and Sacha Gobeyn led the writing of the manuscript. All authors contributed critically to the drafts and gave final approval for publication.
