# Supplementary material for: Assessing the suitable habitat for reintroduction of brown trout (Salmo trutta forma fario) in a lowland river: A modeling approach
Source: Ecol Evol. 2018 Apr 27;8(10):5191–205. doi: 10.1002/ece3.4022 (PMC5980458; doi:10.1002/ece3.4022)
Supplement: Supplementary file 1 [file ECE3-8-5191-s001.docx]

**Supportive information A: Data processing**

The abundance data of brown trout were summed for every point in time and space, and afterwards transformed to presence-absence data (abundance > 0 is equal to presence, abundance = 0 is equal to absence). In order to explore and identify outliers for the abiotic data, box plots, histograms and dot plots were inspected (Figures A1 to A4). Based on these plots, outliers (sampling points) were identified. This was done by checking all values outside the 5 and 95 percentile (Table A1) and by checking the extreme values on the dot plots and distributions in the box plots and histograms.

The sampling points were checked one by one for their validity based on one or more of these criteria:

1. Did the values reported for the point coincide with the metadata?
2. Was the mass balance (e.g. total nitrogen) correct?
3. Did the information coincide with available GIS information?
4. Did the values coincide with the physical boundary conditions?

In addition, all points with a river width of 0 were omitted, unless there were values reported for the width of the measured transect. In this case, this value was used as an estimate of width. After these preprocessing steps, the correlation between the abiotic variables was calculated (Table A2) and the presence-absence of the species was plotted as a function of the possible explanatory variable values (see Figure A5 to A10, upper panel) and an estimate of the habitat suitability curves were obtained (see Figure A5 to A10, lower panel). As explained in section 2.3, these habitat curves were defined by four parameters representing the range and optimal range in which a species is assumed to be present. The lower and upper boundary was estimated by taking the lower and upper value for which the species was observed. These values were calculated several times to account for uncertainty, by bootstrapping the abiotic data for the species presence records a number of times (200 times). The median for the lower and upper value of the bootstraps was taken as the final value for parameters a_1_ and a_4_. The values for the parameters determining the optimal range were estimated in a similar manner, only the 25 and 75 percentiles of the observed distribution for which a species was observed as present was calculated. For the categorical and binary variables, a suitability index value per class was assigned by dividing the relative share of the class for which the species was observed as present by the relative share of the class in the data. Afterwards, this was normalized with the maximum value, so a value between 0 and 1 was obtained for the suitability index for every class. Also here, bootstrapping was applied on the presence data.


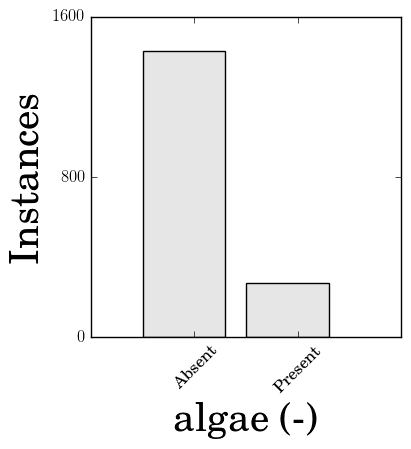

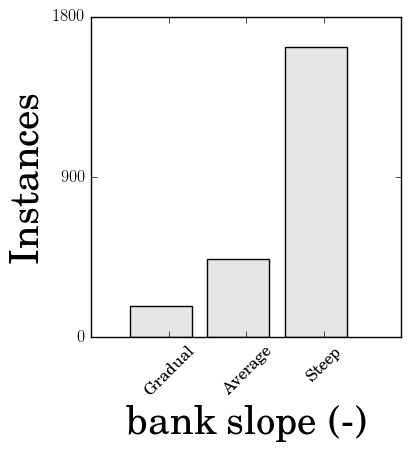

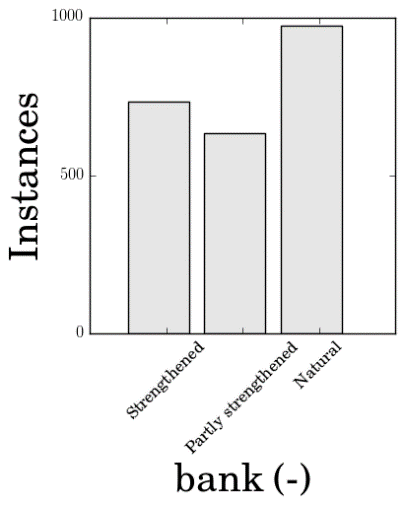

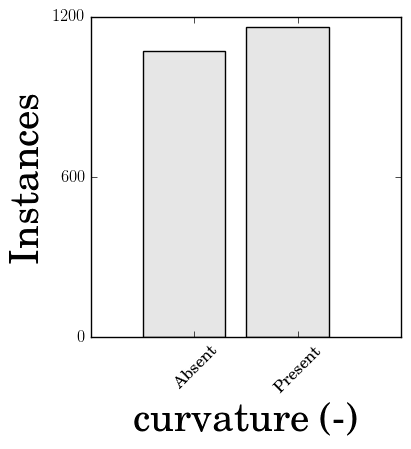

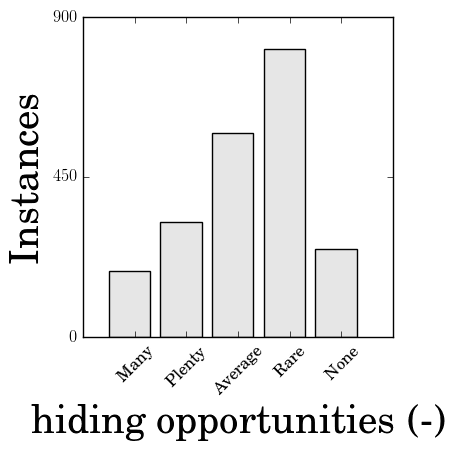

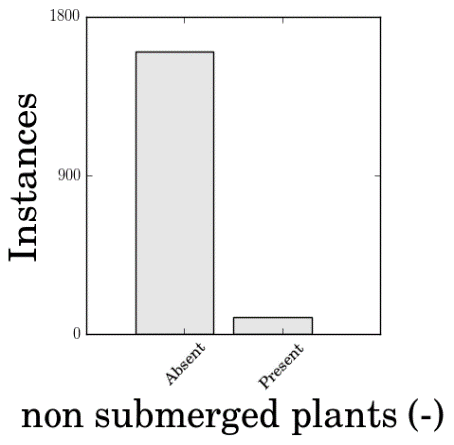

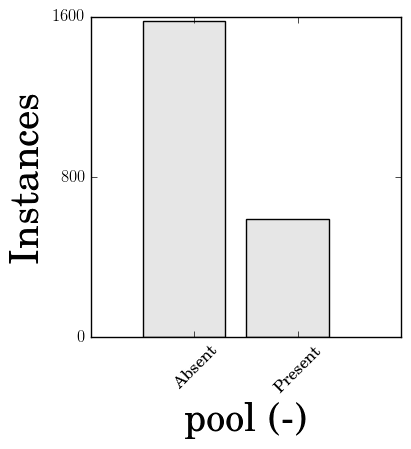

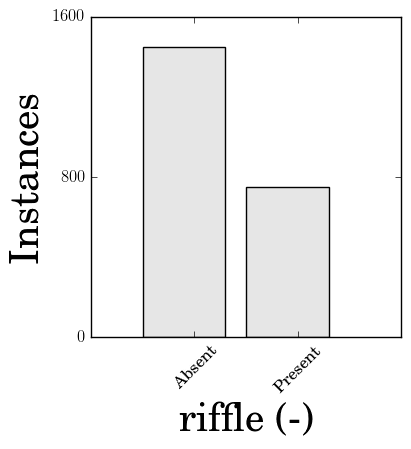

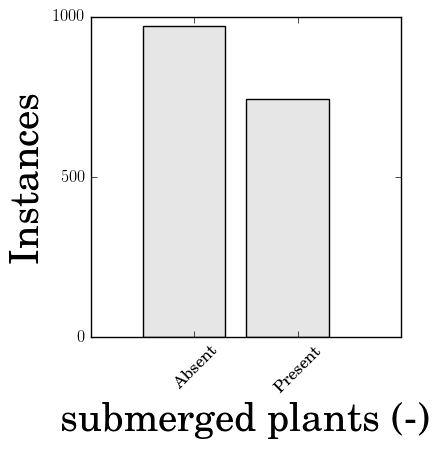

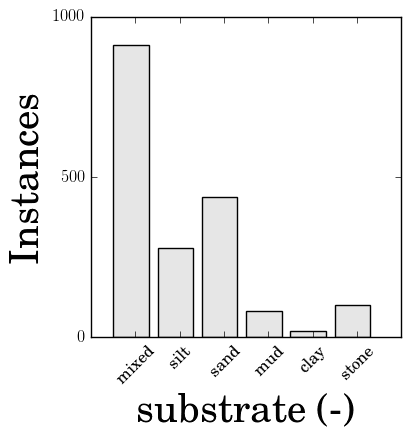


**Figure A1: Instances in every class for algae, bank slope, bank, curvature, hiding opportunities, non-submerged plants, pool, riffle, submerged plants and substrate.**


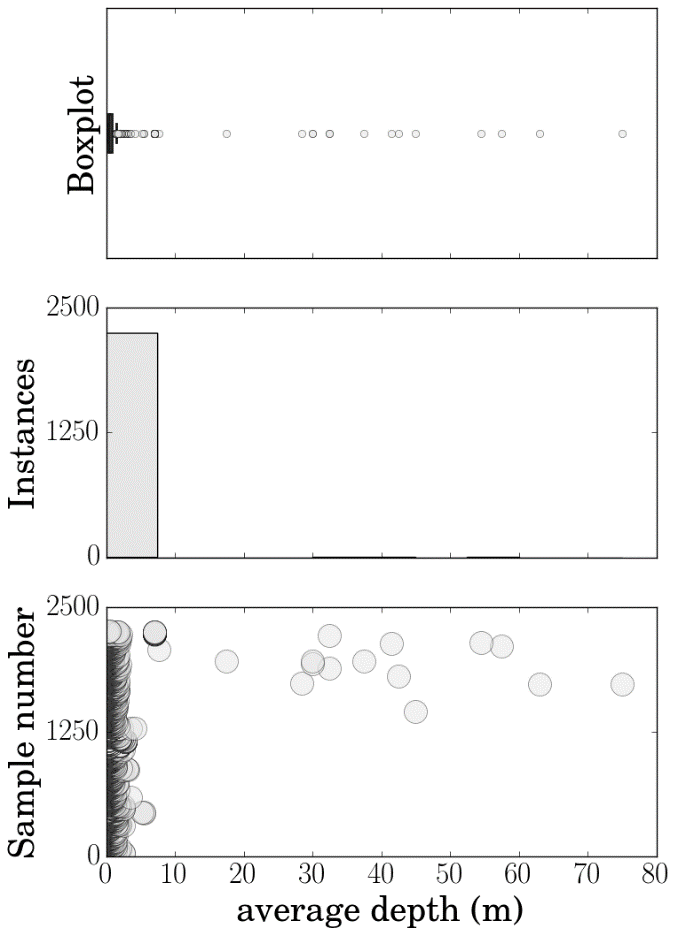

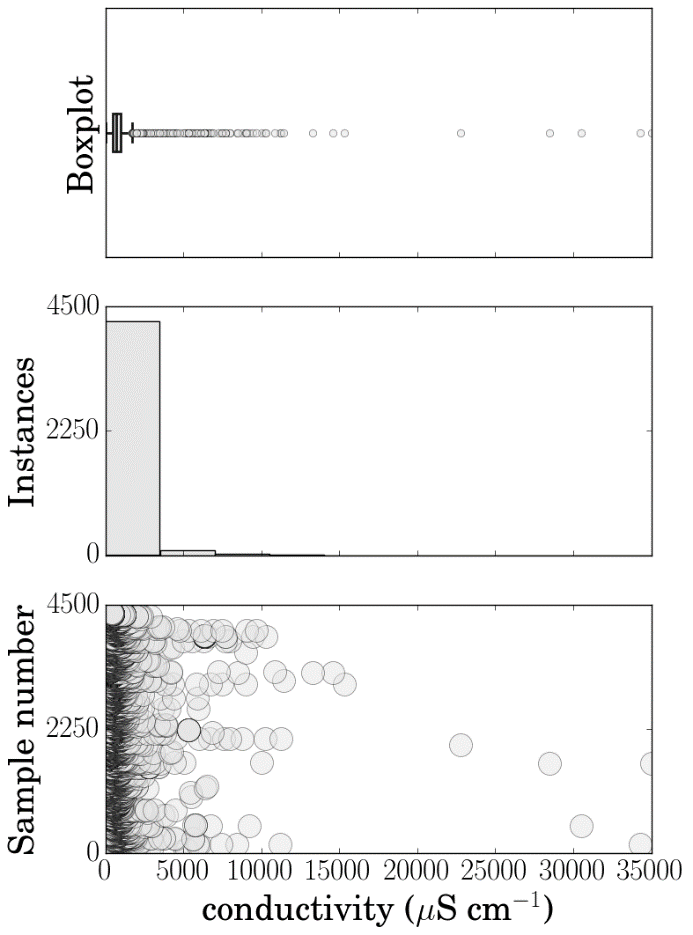

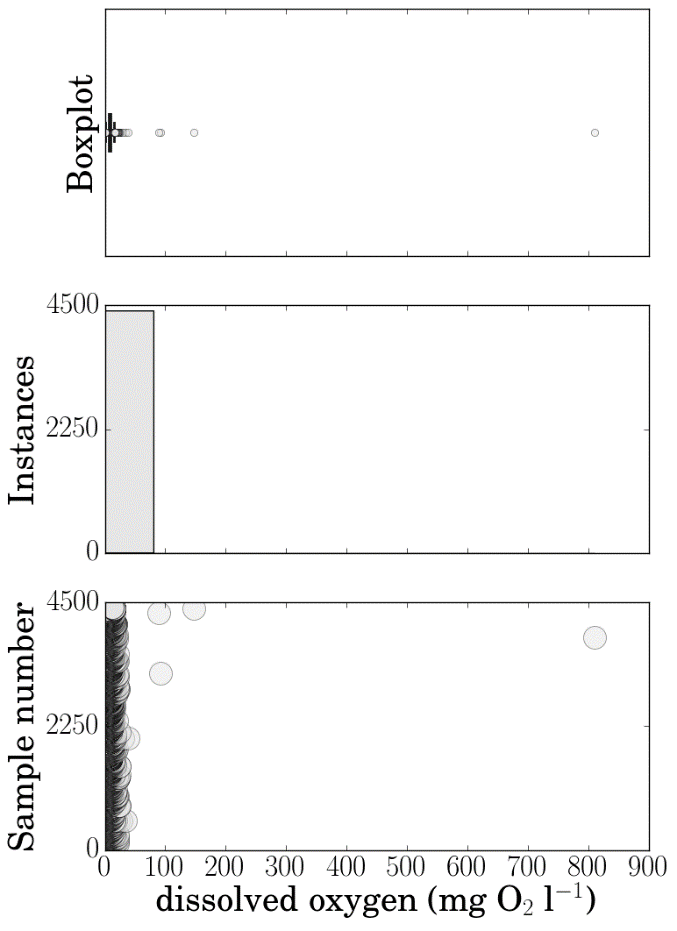

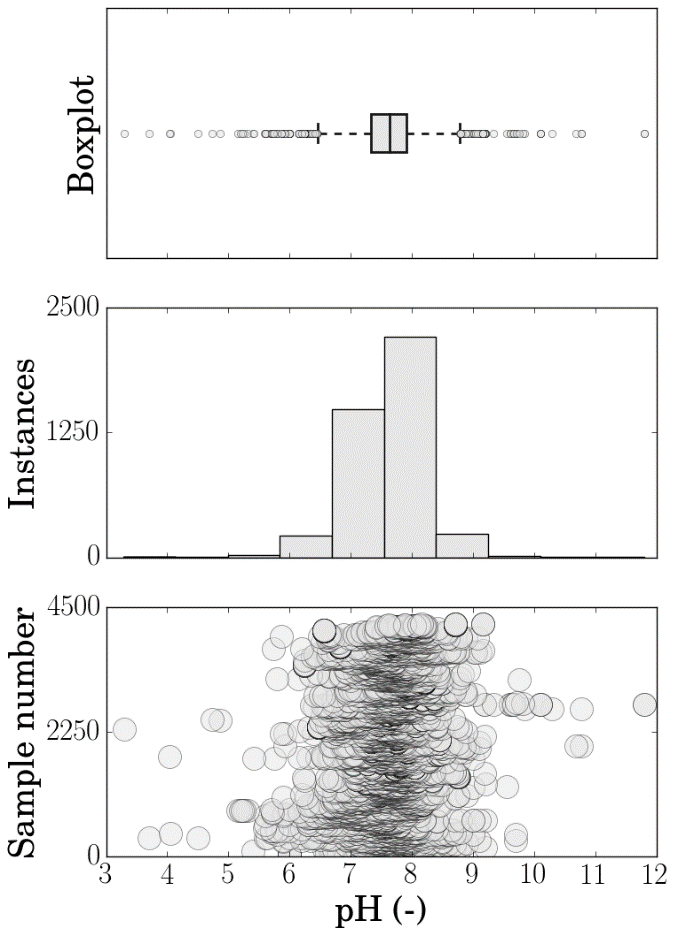


**Figure A2: Boxplot, histogram and dot plot for average depth, conductivity, dissolved oxygen and pH.**


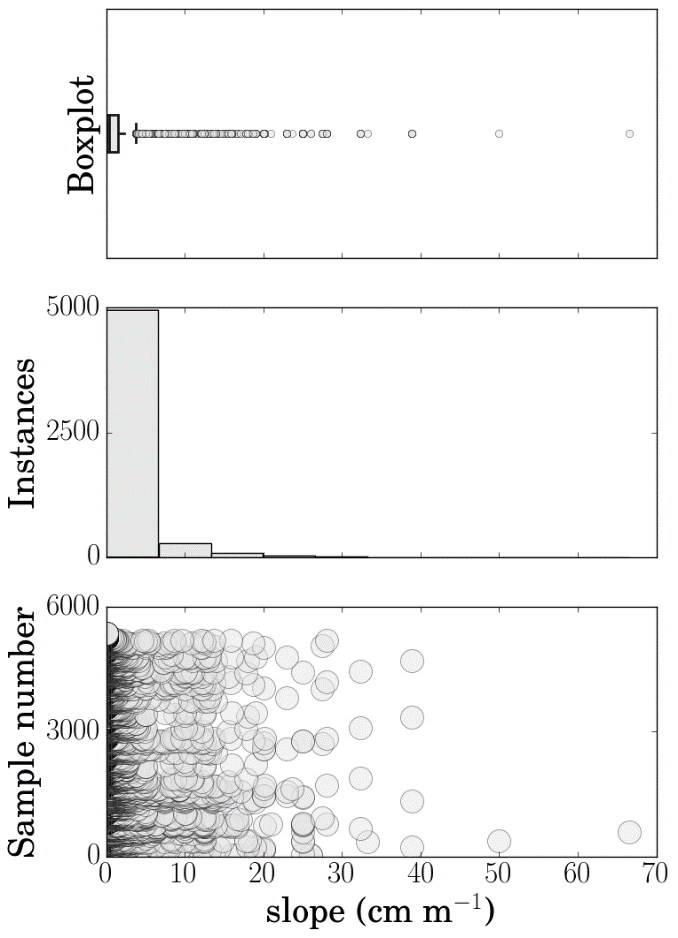

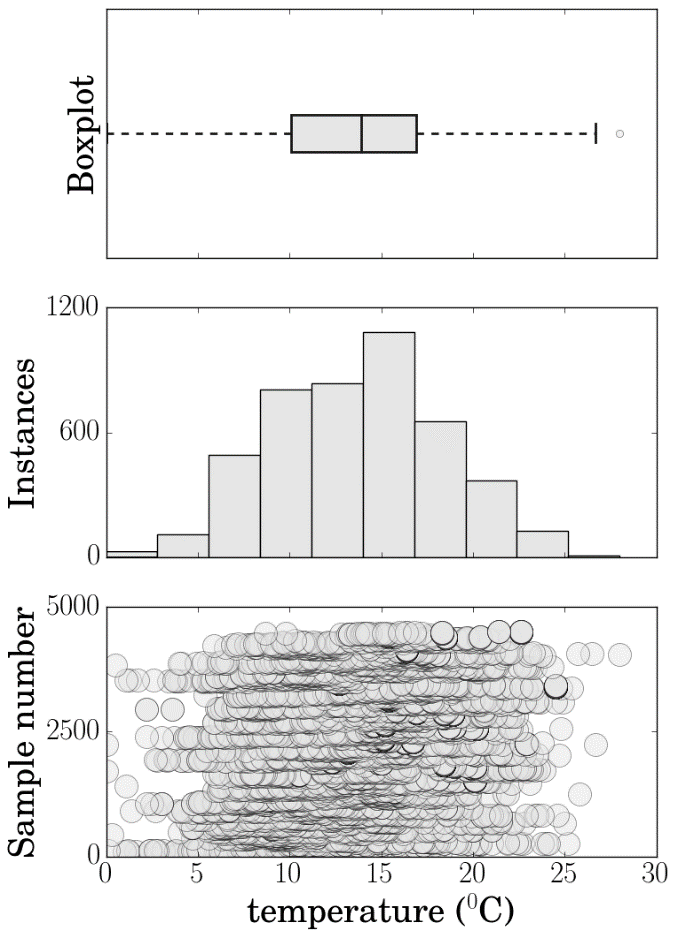

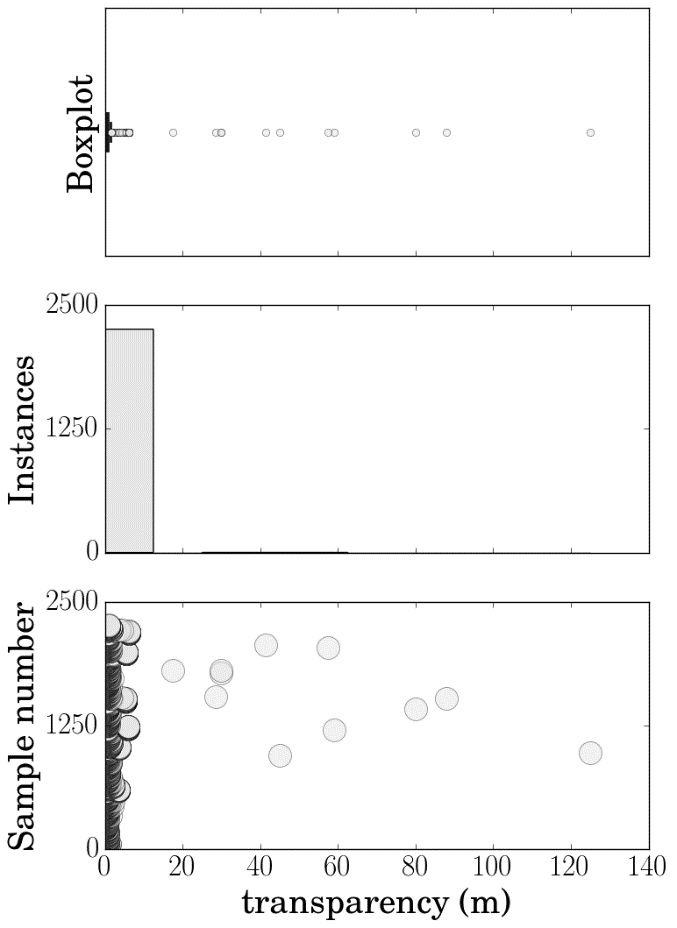

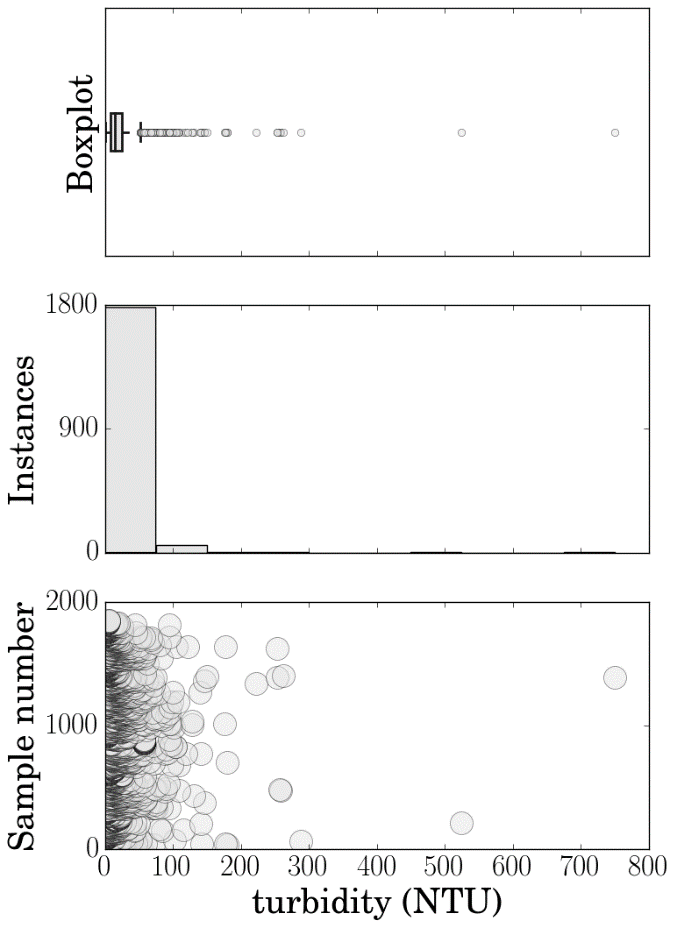


**Figure A3: Boxplot, histogram and dot plot for slope, temperature, transparency, turbidity.**


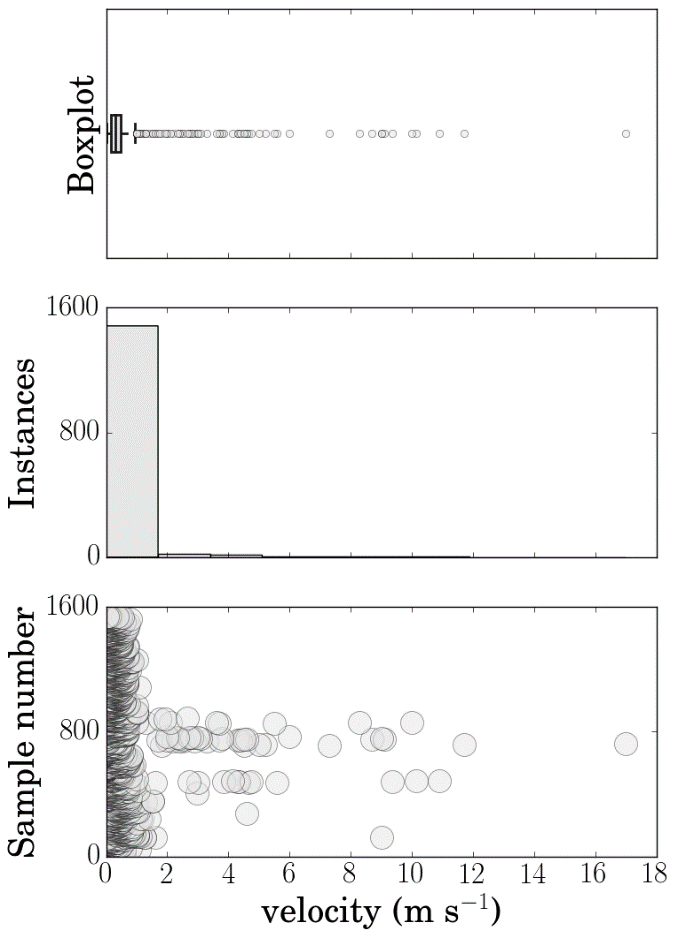

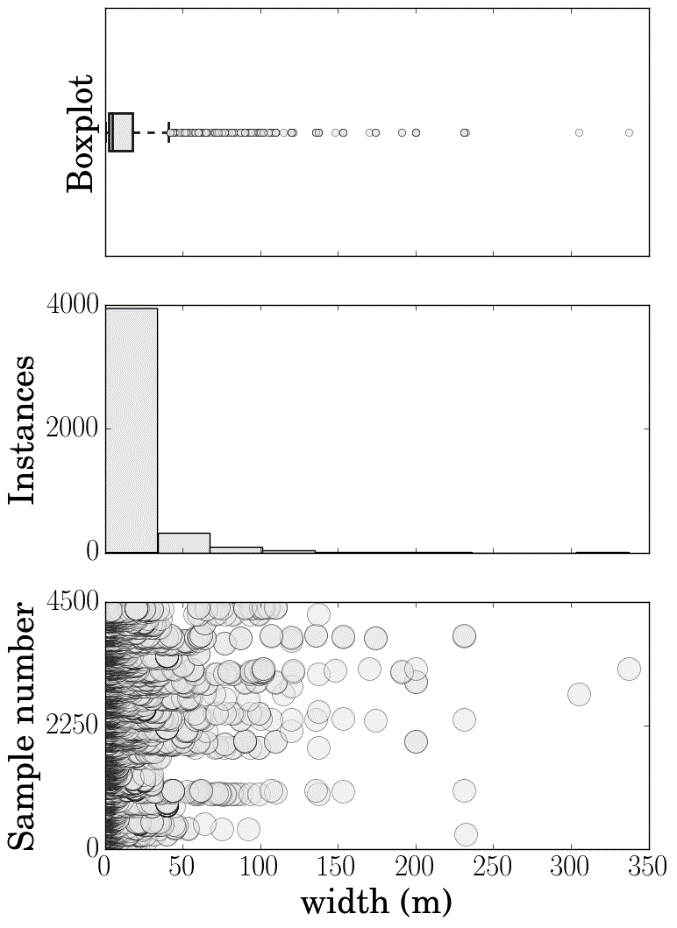


**Figure A4: Boxplot, histogram and dot plot for velocity and width.**

**Table A1: Statistics for variables (units: see Table 1, main manuscript).**

| **variable** | **# samples** | **mean** | **standard deviation** | **minimum** | **25%** | **50%** | **75%** | **maximum** |
| --- | --- | --- | --- | --- | --- | --- | --- | --- |
| algae | 1704 |  |  | 0 | 0.0 | 0.0 | 0.0 | 1.0 |
| area | Omitted from analysis: area was related to the sampling area, which is independent of the species presence-absence. | | | | | | | |
| average depth | 2244 | 0.67 | 0.84 | 0.02 | 0.3 | 0.5 | 0.8 | 7.6 |
| bank | 2341 |  |  | 1 | 1.0 | 2.0 | 3.0 | 3.0 |
| bank slope | 2253 |  |  | 1 | 2.0 | 3.0 | 3.0 | 3.0 |
| barriers | Omitted from analysis: not relevant for management (migration barriers are being removed in the Zwalm). | | | | | | | |
| brackish | Omitted from analysis: all considered systems in this study were freshwater systems. | | | | | | | |
| conductivity | 4341 | 951.62 | 1,470.23 | 5 | 473.0 | 732.0 | 968.0 | 35000.0 |
| curvature | 2238 |  |  | 0 | 0.0 | 1.0 | 1.0 | 1.0 |
| dissolved oxygen | 4380 | 8.02 | 3.26 | 0 | 6.0 | 8.2 | 9.8 | 23.7 |
| distance from spring | Pooled variable not directly indicating the cause of presence-absence were omitted from the analysis. | | | | | | | |
| hiding opportunities | 2139 |  |  | 1 | 3.0 | 3.0 | 4.0 | 5.0 |
| land use | Only variables coupled to direct pressures were considered. | | | | | | | |
| length | Omitted from analysis: length was related to the sampling area, which is independent of the species presence-absence. | | | | | | | |
| non submerged plants | 1703 |  |  | 0 | 0.0 | 0.0 | 0.0 | 1.0 |
| pH | 4193 | 7.60 | 0.58 | 3.29 | 7.3 | 7.6 | 7.9 | 11.8 |
| pool | 2173 |  |  | 0 | 0.0 | 0.0 | 1.0 | 1.0 |
| riffle | 2204 |  |  | 0 | 0.0 | 0.0 | 1.0 | 1.0 |
| slope | 5365 | 1.78 | 4.01 | 0 | 0.0 | 0.4 | 1.5 | 66.6 |
| submerged plants | 1715 |  |  | 0 | 0.0 | 0.0 | 1.0 | 1.0 |
| substrate | 1837 |  |  | 0 | 0.0 | 1.0 | 2.0 | 5.0 |
| temperature | 4503 | 13.68 | 4.60 | 0.1 | 10.1 | 13.9 | 16.9 | 28.0 |
| tidal | Omitted from analysis: al considered systems in this study were non-tidal | | | | | | | |
| transparency | 2252 | 0.86 | 1.21 | 0 | 0.3 | 0.5 | 0.8 | 6.2 |
| turbidity | 1847 | 22.89 | 33.48 | 0.82 | 8.3 | 14.6 | 25.8 | 750.0 |
| velocity | 1537 | 0.48 | 0.98 | 0 | 0.2 | 0.3 | 0.5 | 11.7 |
| water depth | Omitted from analysis: insufficient metadata. | | | | | | | |
| width | 4393 | 13.90 | 23.52 | 0.25 | 2.3 | 4.9 | 18.0 | 337.0 |
| width transect | Omitted from analysis: width transect was related to the sampling area, which is independent of the species presence-absence. | | | | | | | |

**Table A2: Spearman rank correlation (cond. = conductivity, opp. = opportunities, sub = submerged, temp. = temperature, turb. = turbiditiy, vel = velocity). For units, see Table 1.**

| **variable** | **algae** | **average depth** | **bank** | **bank slope** | **cond.** | **curvature** | **dissolved oxygen** | **hiding opp.** | **non sub. plants** | **pH** | **pool** | **riffle** | **slope** | **sub. plants** | **substrate** | **temp.** | **transp.** | **turb.** | **vel.** | **width** |
| --- | --- | --- | --- | --- | --- | --- | --- | --- | --- | --- | --- | --- | --- | --- | --- | --- | --- | --- | --- | --- |
| algae | 1.00 | 0.06 | -0.01 | 0.08 | 0.00 | 0.05 | 0.02 | -0.03 | 0.09 | 0.12 | 0.01 | 0.03 | -0.04 | 0.12 | -0.03 | 0.19 | 0.08 | -0.19 | -0.04 | 0.01 |
| average depth | 0.06 | 1.00 | -0.11 | 0.09 | 0.07 | 0.00 | 0.02 | -0.02 | 0.11 | 0.06 | -0.09 | -0.08 | -0.41 | 0.14 | 0.00 | 0.10 | 0.76 | -0.09 | 0.10 | 0.67 |
| bank | -0.01 | -0.11 | 1.00 | -0.22 | -0.19 | 0.19 | 0.05 | -0.42 | -0.03 | -0.16 | 0.23 | 0.16 | 0.27 | 0.03 | -0.01 | -0.14 | -0.25 | 0.06 | -0.09 | -0.39 |
| bank slope | 0.08 | 0.09 | -0.22 | 1.00 | 0.19 | -0.08 | -0.06 | 0.27 | -0.01 | 0.08 | -0.20 | -0.12 | -0.07 | -0.05 | -0.02 | 0.11 | 0.11 | 0.01 | 0.03 | 0.14 |
| conductivity | 0.00 | 0.07 | -0.19 | 0.19 | 1.00 | -0.10 | -0.20 | 0.22 | -0.04 | 0.27 | -0.16 | -0.14 | -0.03 | -0.08 | -0.05 | -0.01 | -0.07 | 0.18 | -0.06 | 0.16 |
| curvature | 0.05 | 0.00 | 0.19 | -0.08 | -0.10 | 1.00 | 0.06 | -0.23 | -0.01 | -0.07 | 0.35 | 0.36 | 0.30 | 0.01 | -0.07 | -0.13 | -0.09 | 0.04 | 0.10 | -0.30 |
| dissolved oxygen | 0.02 | 0.02 | 0.05 | -0.06 | -0.20 | 0.06 | 1.00 | -0.12 | -0.05 | 0.23 | 0.13 | 0.12 | 0.03 | 0.03 | 0.00 | -0.16 | 0.09 | -0.12 | 0.17 | -0.08 |
| hiding opportunities | -0.03 | -0.02 | -0.42 | 0.27 | 0.22 | -0.23 | -0.12 | 1.00 | -0.07 | 0.13 | -0.44 | -0.36 | -0.30 | -0.23 | 0.03 | 0.10 | 0.14 | 0.04 | -0.11 | 0.23 |
| non sub. plants | 0.09 | 0.11 | -0.03 | -0.01 | -0.04 | -0.01 | -0.05 | -0.07 | 1.00 | -0.01 | 0.01 | -0.04 | -0.08 | 0.20 | 0.05 | 0.16 | 0.07 | -0.04 | -0.04 | 0.06 |
| pH | 0.12 | 0.06 | -0.16 | 0.08 | 0.27 | -0.07 | 0.23 | 0.13 | -0.01 | 1.00 | -0.03 | -0.03 | -0.20 | -0.10 | -0.11 | 0.15 | 0.13 | -0.09 | -0.01 | 0.15 |
| pool | 0.01 | -0.09 | 0.23 | -0.20 | -0.16 | 0.35 | 0.13 | -0.44 | 0.01 | -0.03 | 1.00 | 0.72 | 0.38 | -0.01 | -0.08 | -0.19 | -0.18 | 0.02 | 0.20 | -0.32 |
| riffle | 0.03 | -0.08 | 0.16 | -0.12 | -0.14 | 0.36 | 0.12 | -0.36 | -0.04 | -0.03 | 0.72 | 1.00 | 0.44 | -0.05 | -0.07 | -0.21 | -0.15 | 0.06 | 0.24 | -0.35 |
| slope | -0.04 | -0.41 | 0.27 | -0.07 | -0.03 | 0.30 | 0.03 | -0.30 | -0.08 | -0.20 | 0.38 | 0.44 | 1.00 | -0.10 | -0.09 | -0.42 | -0.56 | 0.30 | 0.26 | -0.65 |
| Sub. plants | 0.12 | 0.14 | 0.03 | -0.05 | -0.08 | 0.01 | 0.03 | -0.23 | 0.20 | -0.10 | -0.01 | -0.05 | -0.10 | 1.00 | 0.07 | 0.14 | 0.09 | -0.04 | -0.09 | 0.06 |
| substrate | -0.03 | 0.00 | -0.01 | -0.02 | -0.05 | -0.07 | 0.00 | 0.03 | 0.05 | -0.11 | -0.08 | -0.07 | -0.09 | 0.07 | 1.00 | 0.00 | 0.04 | 0.01 | -0.06 | 0.06 |
| temperature | 0.19 | 0.10 | -0.14 | 0.11 | -0.01 | -0.13 | -0.16 | 0.10 | 0.16 | 0.15 | -0.19 | -0.21 | -0.42 | 0.14 | 0.00 | 1.00 | 0.25 | -0.16 | -0.27 | 0.35 |
| transparency | 0.08 | 0.76 | -0.25 | 0.11 | -0.07 | -0.09 | 0.09 | 0.14 | 0.07 | 0.13 | -0.18 | -0.15 | -0.56 | 0.09 | 0.04 | 0.25 | 1.00 | -0.48 | 0.03 | 0.54 |
| turbidity | -0.19 | -0.09 | 0.06 | 0.01 | 0.18 | 0.04 | -0.12 | 0.04 | -0.04 | -0.09 | 0.02 | 0.06 | 0.30 | -0.04 | 0.01 | -0.16 | -0.48 | 1.00 | 0.04 | -0.11 |
| velocity | -0.04 | 0.10 | -0.09 | 0.03 | -0.06 | 0.10 | 0.17 | -0.11 | -0.04 | -0.01 | 0.20 | 0.24 | 0.26 | -0.09 | -0.06 | -0.27 | 0.03 | 0.04 | 1.00 | 0.04 |
| width | 0.01 | 0.67 | -0.39 | 0.14 | 0.16 | -0.30 | -0.08 | 0.23 | 0.06 | 0.15 | -0.32 | -0.35 | -0.65 | 0.06 | 0.06 | 0.35 | 0.54 | -0.11 | 0.04 | 1.00 |


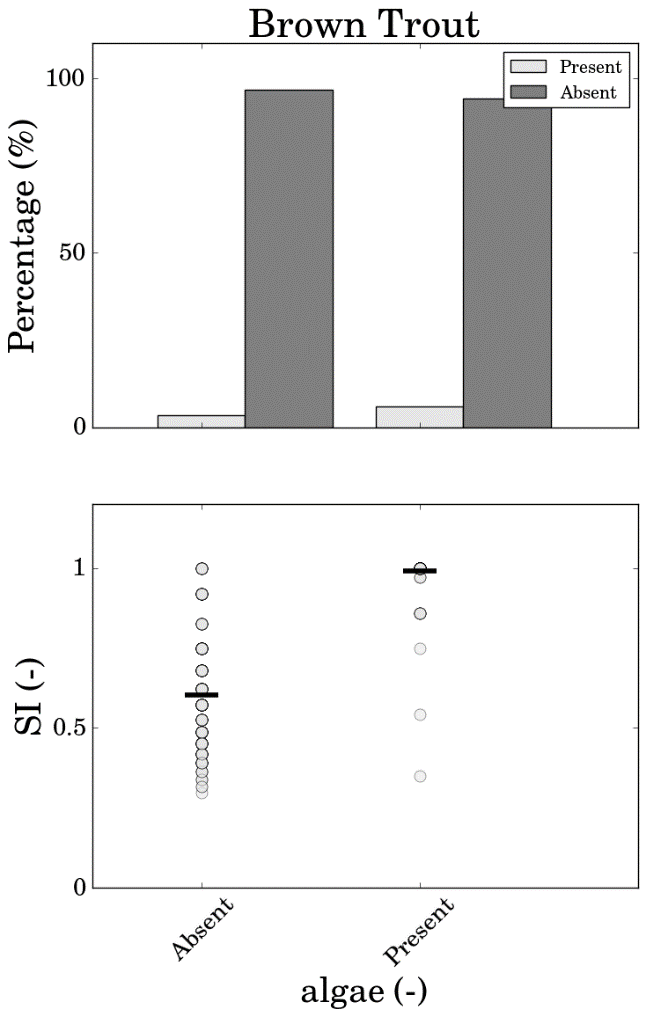

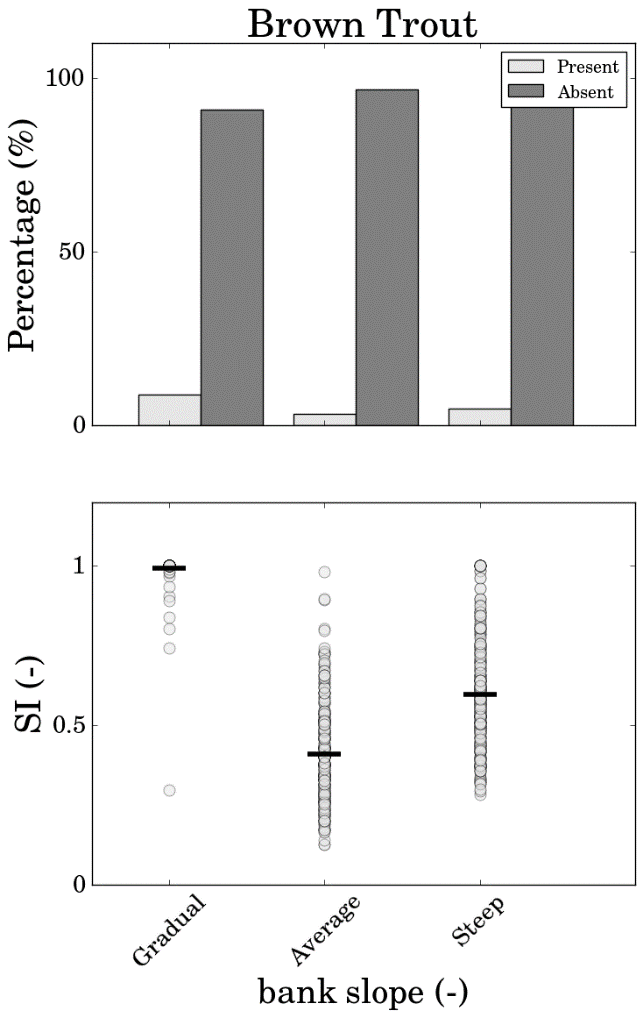

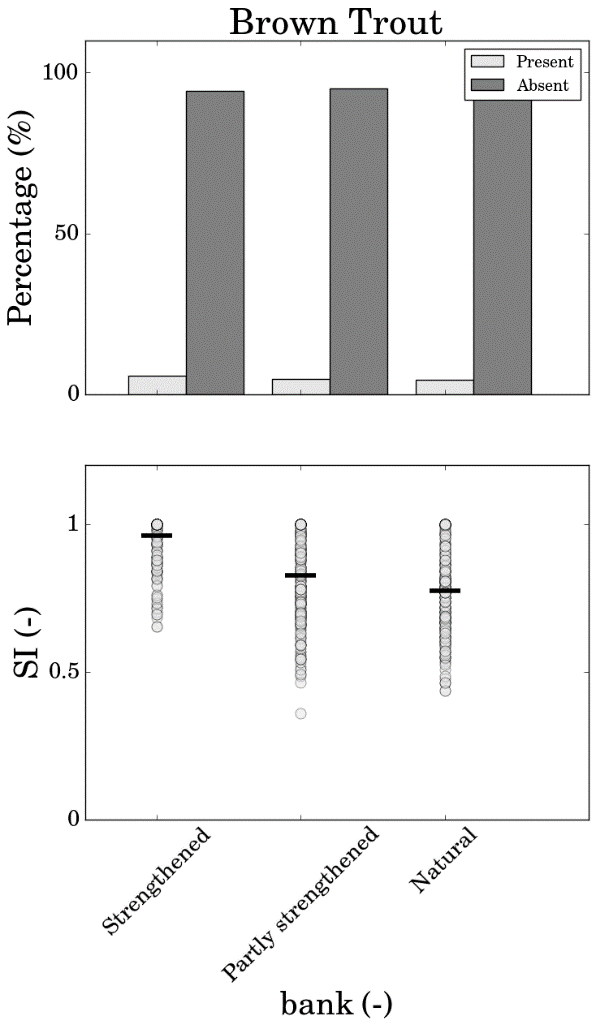

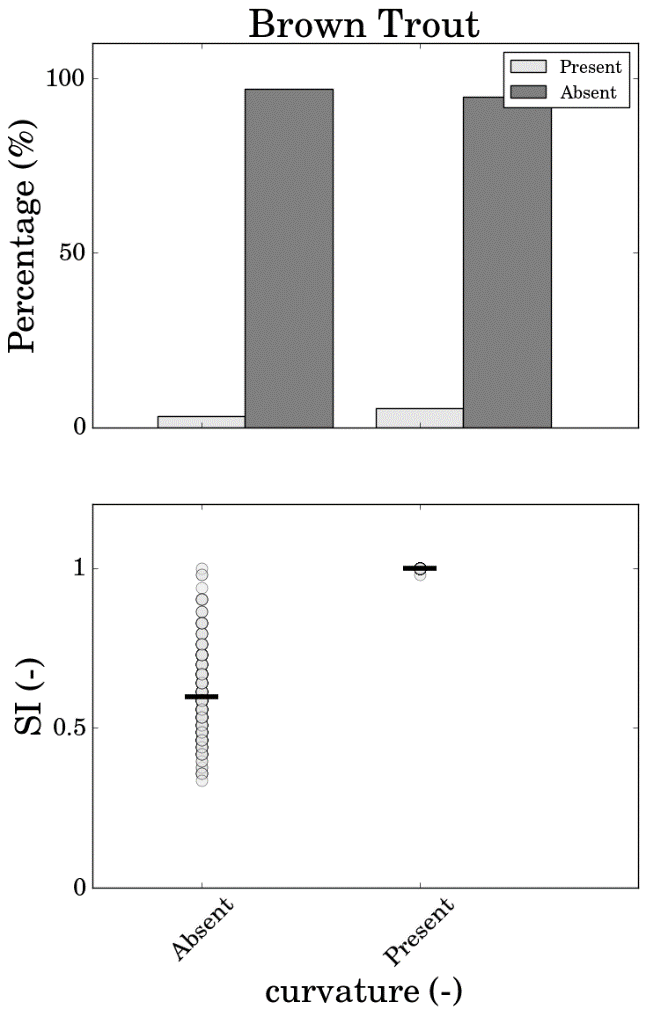


**Figure A5: Presence-absence for every class and estimated SI for categorical and binary variables (algae, bank slope, bank, curvature). Every individual points presents one bootstrap and the median is indicated with the black line.**


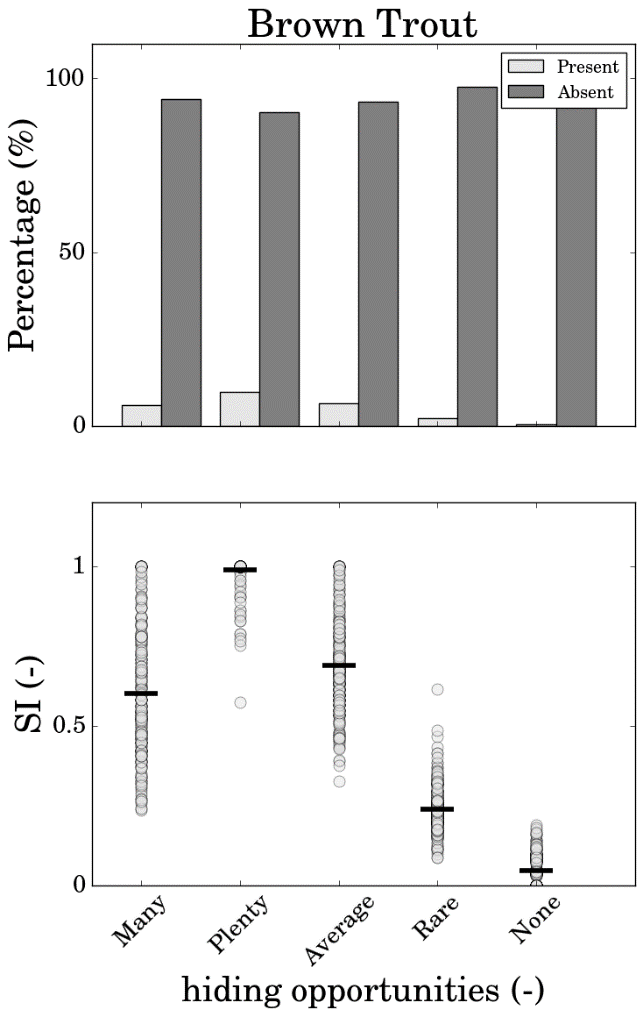

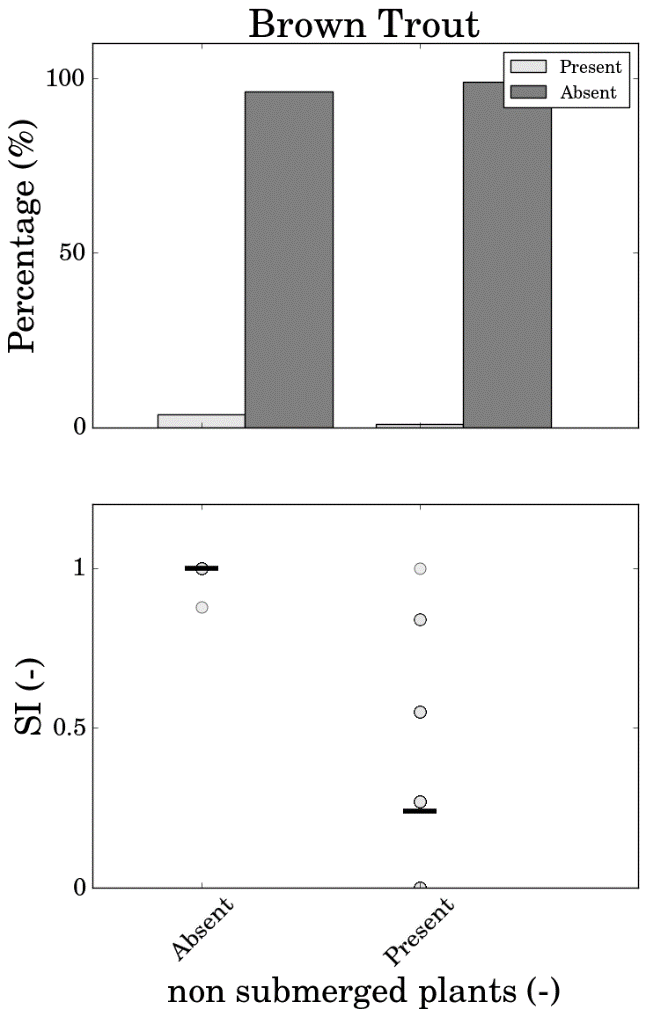

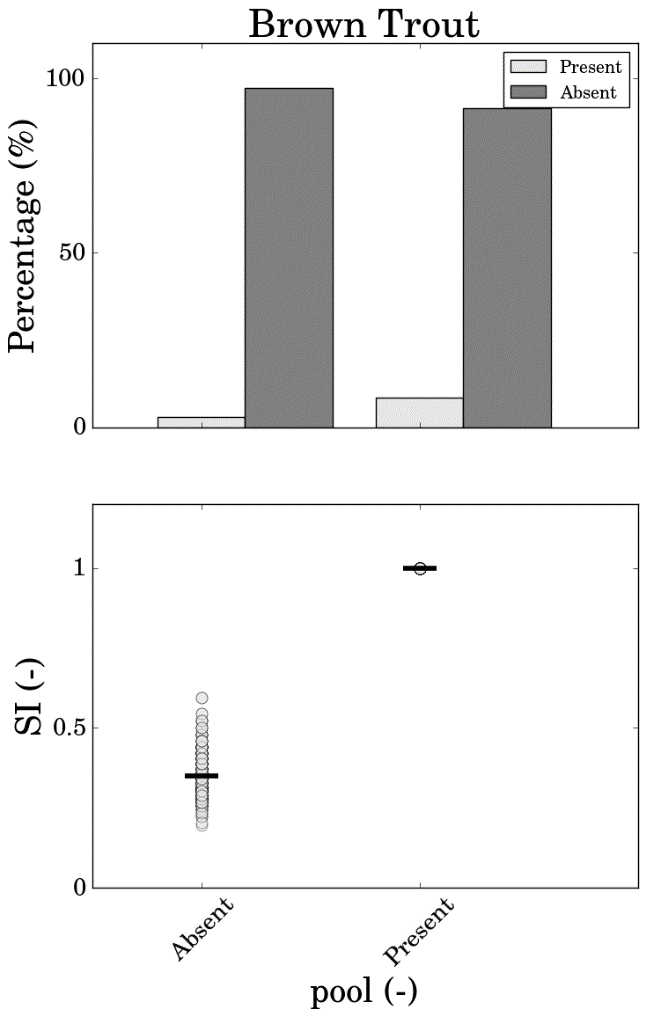

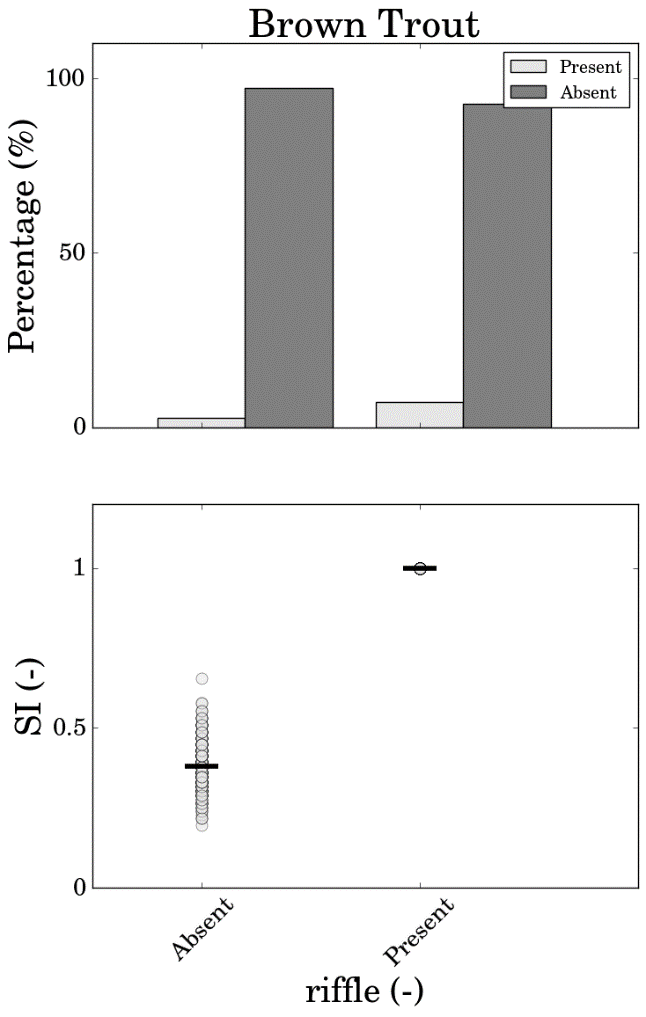


**Figure A6: Presence-absence for every class and estimated SI for categorical and binary variables (hiding opportunities, non-submerged plants, pool and riffle). Every individual points presents one bootstrap and the median is indicated with the black line.**


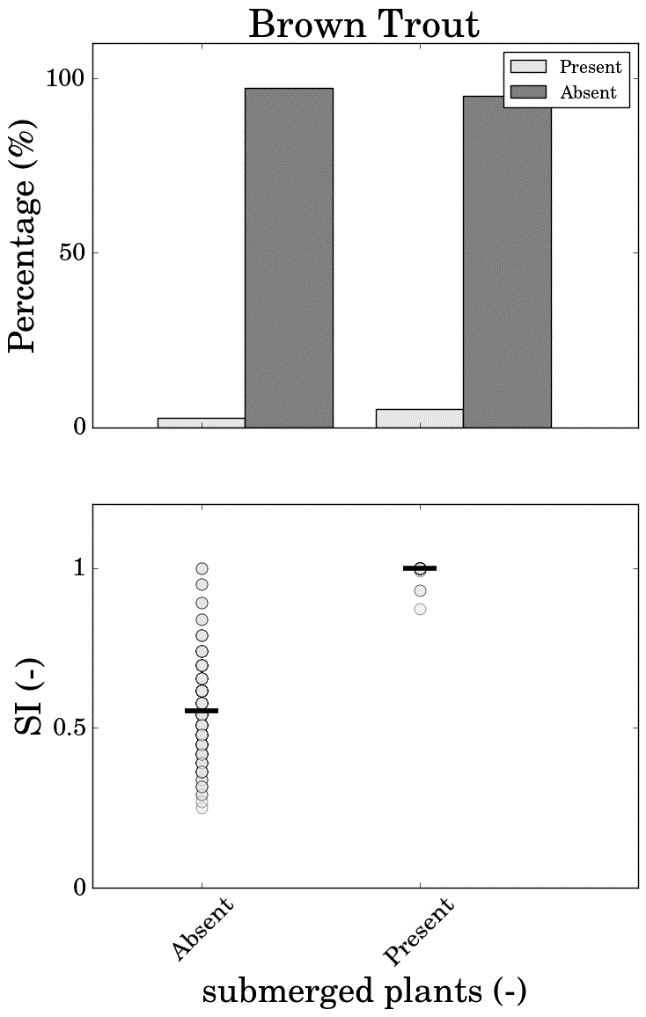

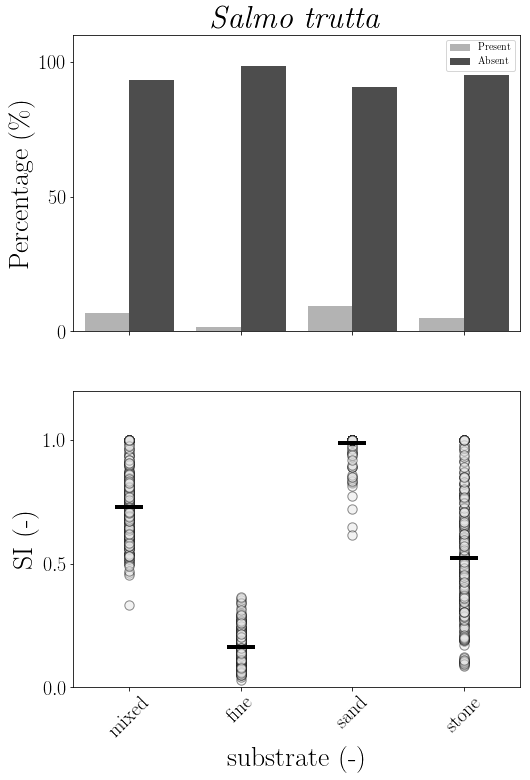


**Figure A7: Presence-absence for every class and estimated SI for categorical and binary variables (submerged plants and substrate). Every individual points presents one bootstrap and the median is indicated with the black line.**


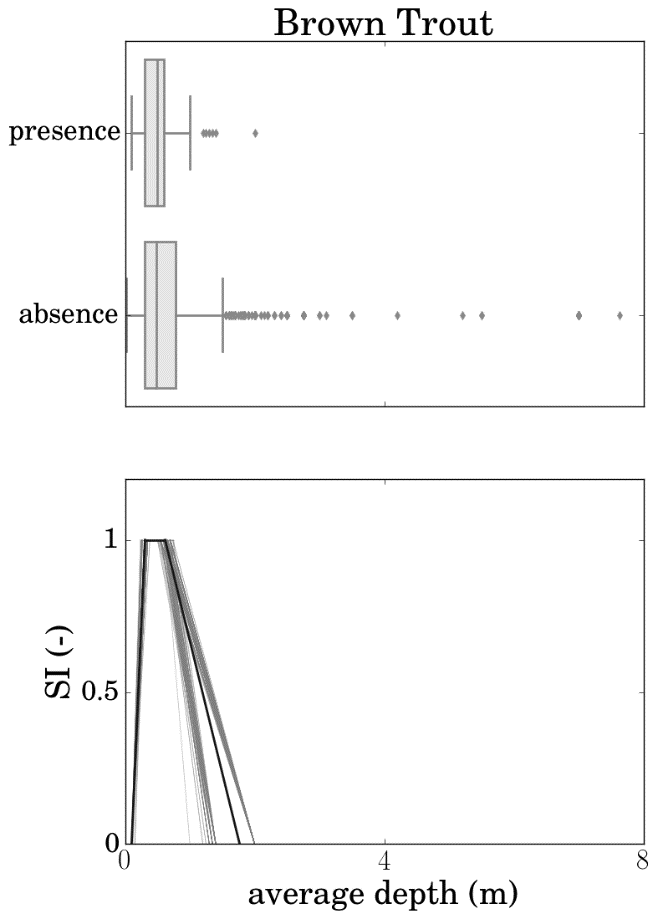

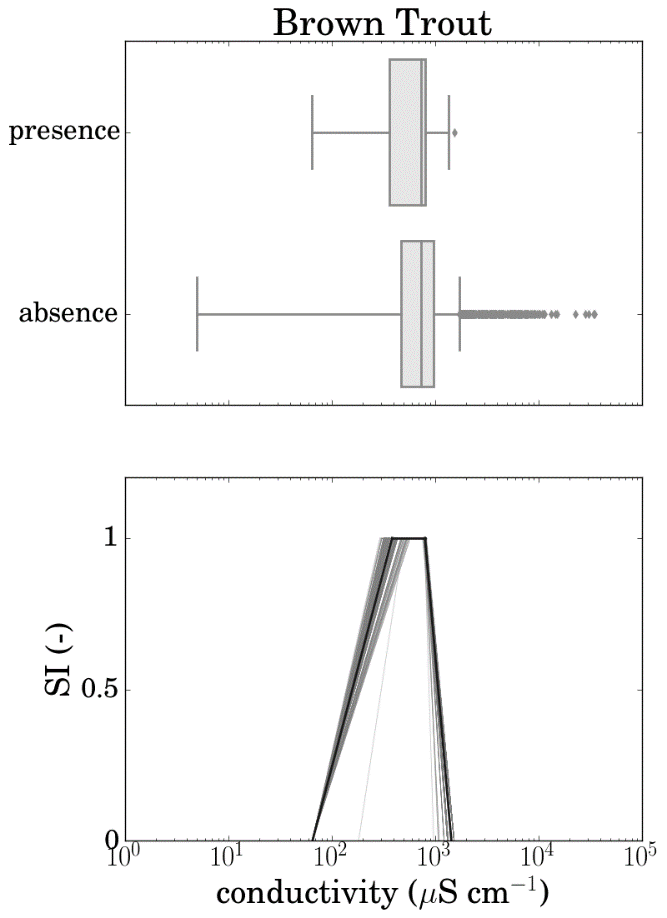

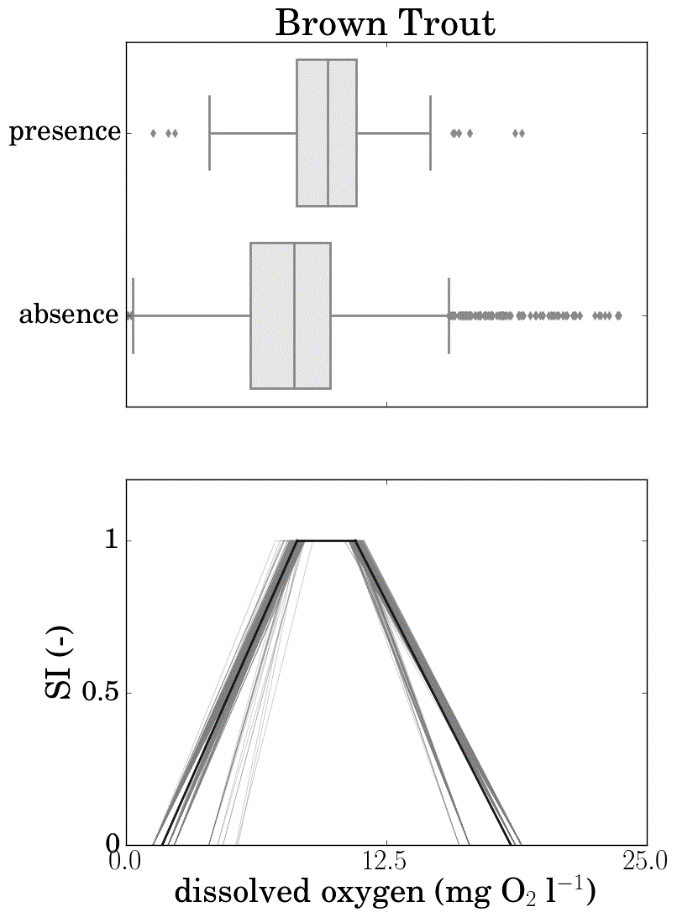

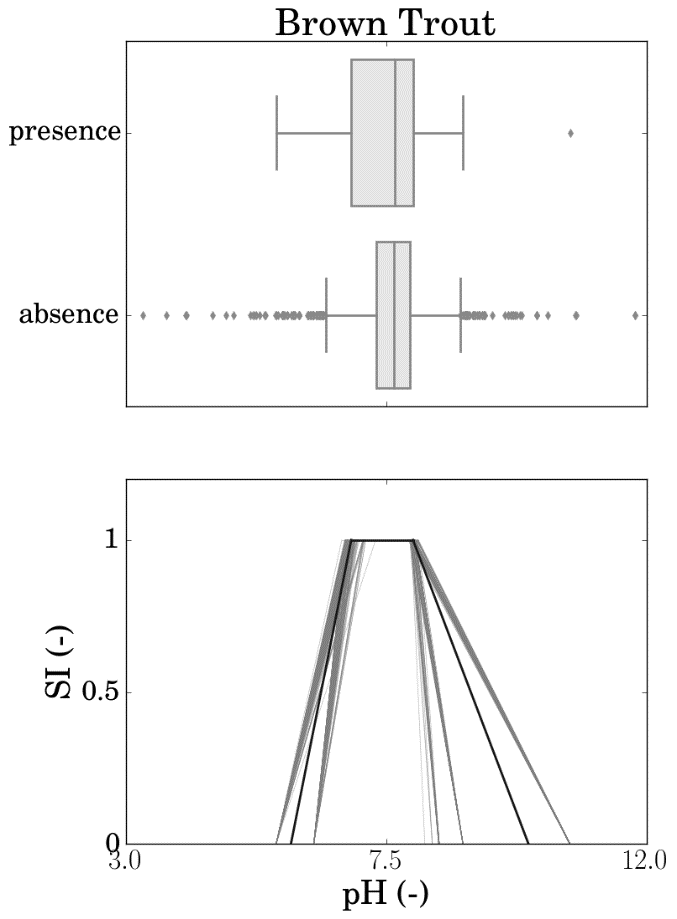


**Figure A8: Boxplot of values for presence and absence of the species and estimated SI for continuous variables (average depth, conductivity, dissolved oxygen and pH). Every individual curve presents one bootstrap and the curve constructed from the median values of a_1_, a_2_, a_3_ and a_4_ is indicated in black.**


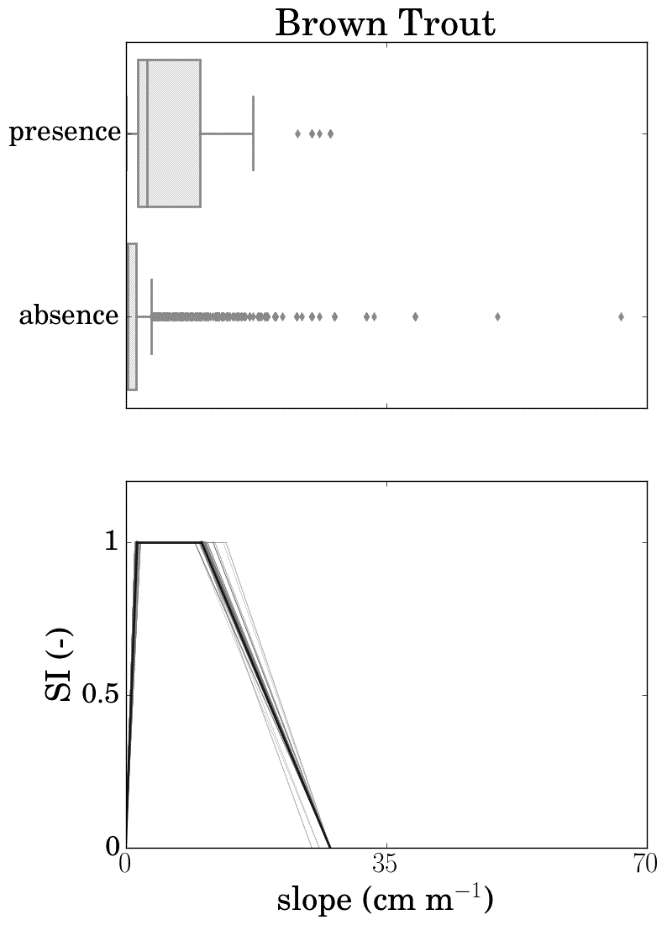

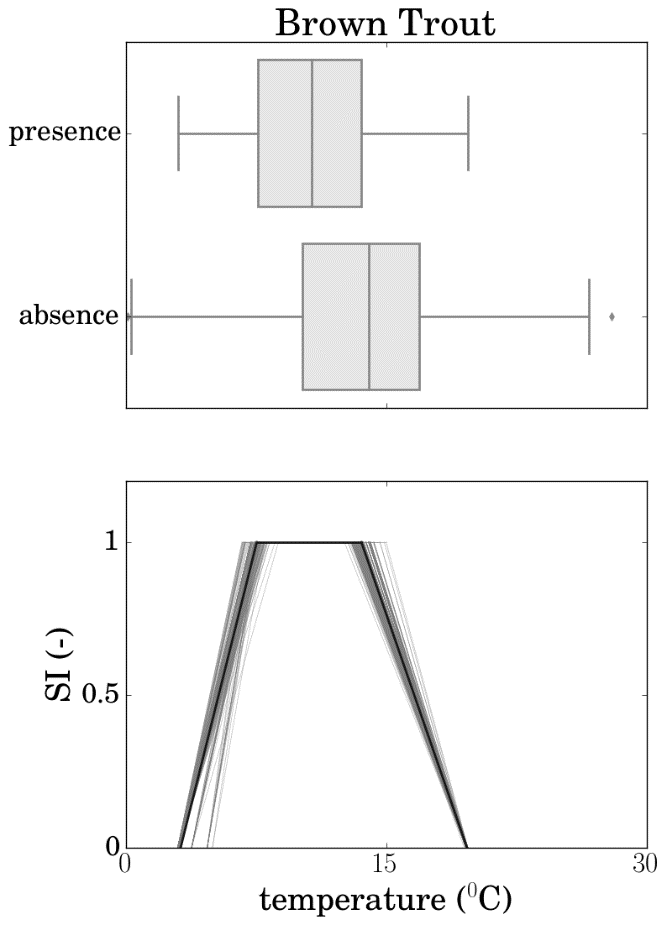


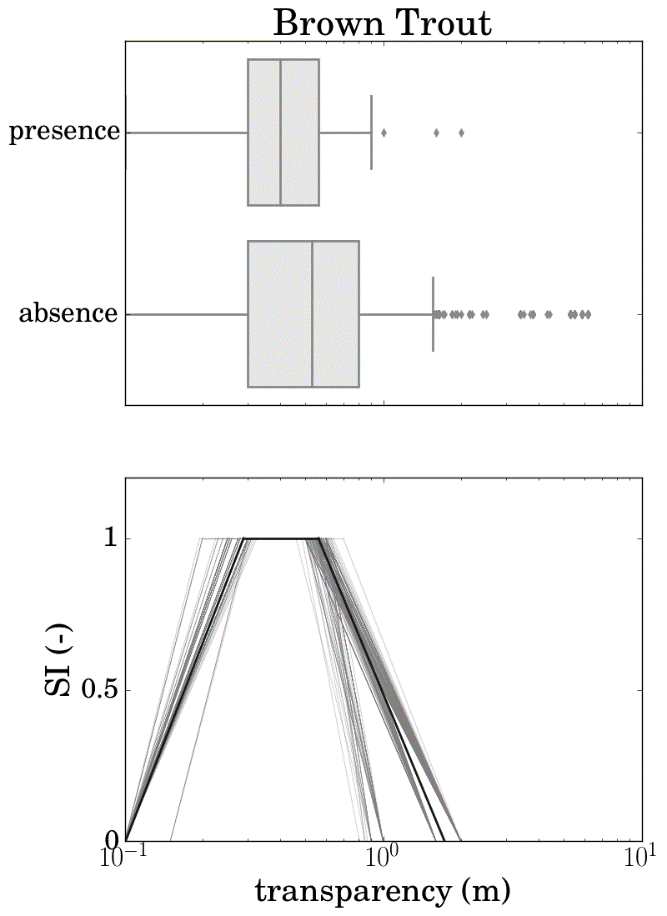

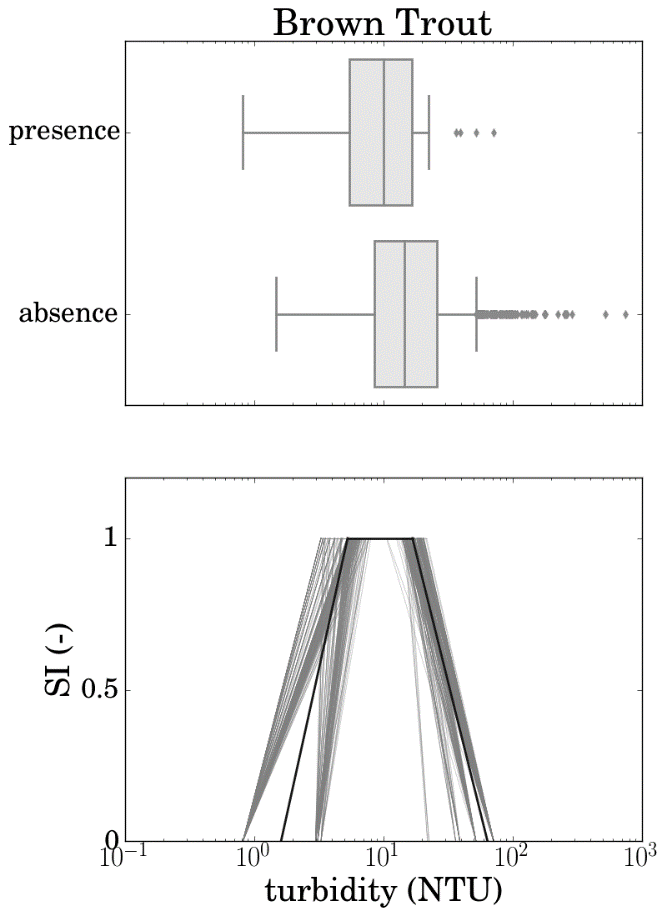


**Figure A9: Boxplot of values for presence and absence of the species and estimated SI for continuous variables (slope, temperature, transparency and turbidity). Every individual curve presents one bootstrap and the curve constructed from the median values of a_1_, a_2_, a_3_ and a_4_ is indicated in black.**


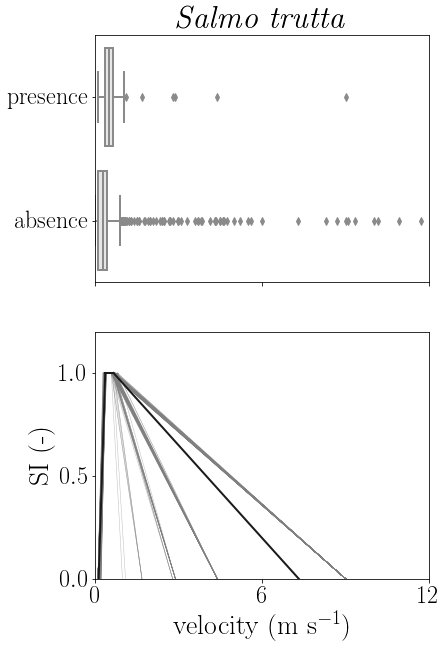

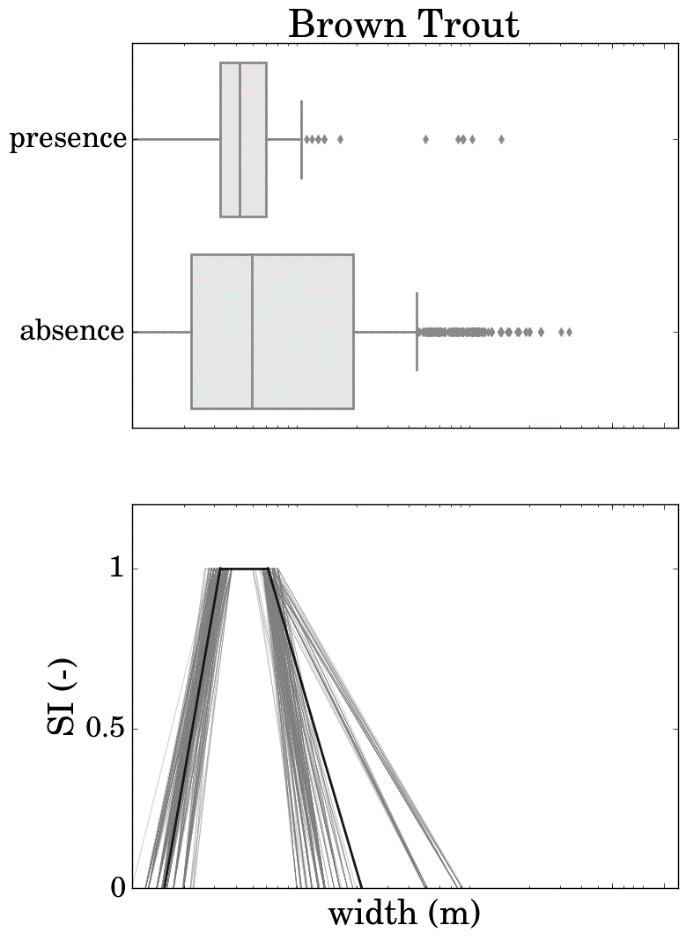


**Figure A10: Boxplot of values for presence and absence of the species and estimated SI for continuous variables (velocity and width). Every individual curve presents one bootstrap and the curve constructed from the median values of a_1_, a_2_, a_3_ and a_4_ is indicated in black.**

**Supportive information B: SDMIT**

The Species Distribution Model Identification Tool (SDMIT) implemented by Gobeyn *et al.* (2017) (https://github.com/Sachagobeyn/SDMIT) was used to optimise the habitat suitability model. In this tool, a simple genetic algorithm (SGA) was used to select the habitat preference curves (HPCs) best explaining the presence-absence of the species. In the remainder of this section, it is explained which objective function and what algorithm (and settings) was used.

*Objective function*

In this paper, we aimed to obtain a number of relatively simple and well-performing models (Ellison 2004) from the defined habitat preference curves. The model complexity and goodness-of-fit with the data were evaluated with the adjusted Akaike Information Criterion (AIC):

$\mathrm{AIC}=n\log\frac{\mathrm{SSE}}{n}+2m+\frac{2m\left( m+1 \right)}{n-m-1}$ (B1)

With $n$, the number of data points, SSE, the sum of squared errors between the simulated habitat suitability index (his) and the presence-absence records. The adjusted AIC was used in order to account for the small number (*i.e.* 50 records) of measurement points.

*Simple genetic algorithm*

An SGA was used to develop an ensemble of models from the defined HPCs. An SGA is a specific implementation of evolutionary algorithms, which are a suite of methods which incorporate elements of structured randomness for search, based on phenoma observed in nature, i.e. selection, mutation and crossover (Goldberg 1989; Maier *et al.* 2014). In general, SGAs are considered as excellent tools to identify a(n) (series of) optimal solution(s) to complex real-world problems, often characterized by much noise (Maier *et al.* 2014).

In this paper, we used the input variable selection (IVS) approach for conceptual species distribution models (SDMs) presented by Gobeyn *et al.* (2017). The authors implemented an SGA, with tournament selection, and one-point crossover and mutation operators to iterate a collection of candidate solutions. These candidate solutions are a number of models defined by a set of HPCs. By testing the fitness of the candidate solutions and applying on fitness-based selection and crossover, one iterates the solution to a (near-)optimal set. The mutation operator allows for exploration of other interesting points of the search space of candidate models. The authors showed that the SGA is suitable for IVS, with the knowledge that one should aim for a number of near-optimal solutions to reflect analysis and data uncertainties. The application of the algorithm requires the definition of a number of hyper parameters. Here, a population size of 32 was chosen, with a selection rate of 0.5, a mutation rate of 0.2 and a crossover rate of 1.0. These values were chosen by following the guidelines of Gibbs, Dandy & Maier (2008) for SGAs and by searching in the vicinity of the determined values. It is important to note that other approaches exist to test hyper parameter values, however, those of Gibbs, Dandy & Maier (2008) were assessed as superior over other approaches by Gibbs, Maier & Dandy (2010).

**Supportive information C: Ensemble size**

In order to account for uncertainties caused by the imperfections in the ecological data and the SGA analysis, the process of model optimisation was repeated a number of times with different sets of the data. This repeated model optimisation generated an ensemble of models (Araujo & New 2007), which was used to reflect prediction uncertainty. An ensemble size had to be selected and this was based on the evolution of the support over the number of generated models. The support was calculated as the number of times a HPC was included in a model over the total number of analysed models. The (Shannon) entropy (Shannon 1948) was computed as an estimate of the uncertainty on the support (Gobeyn *et al.* 2017). By inspecting the stability on the support and its uncertainty (Figure C1), a minimal number of runs was determined, in this case 200.


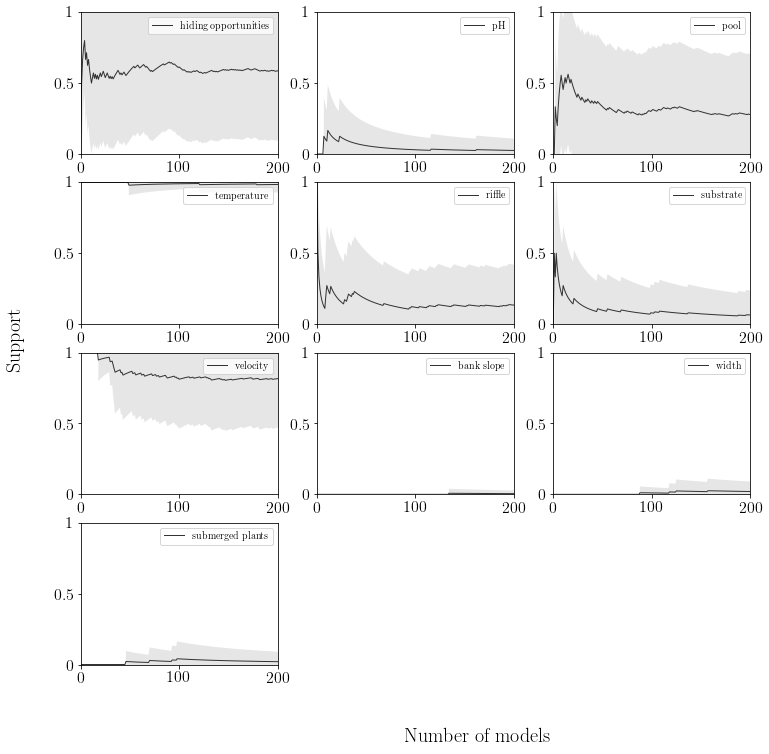


**Figure C1: Evolution of the support of a HPC as a function of the number of analysed models. The support was calculated as the coefficient of the number of times a HPC was included in the model over the number of analysed models. The Shannon entropy was selected as measure for uncertainty (light grey).**

**Supportive information D: Analysis for juveniles**

**
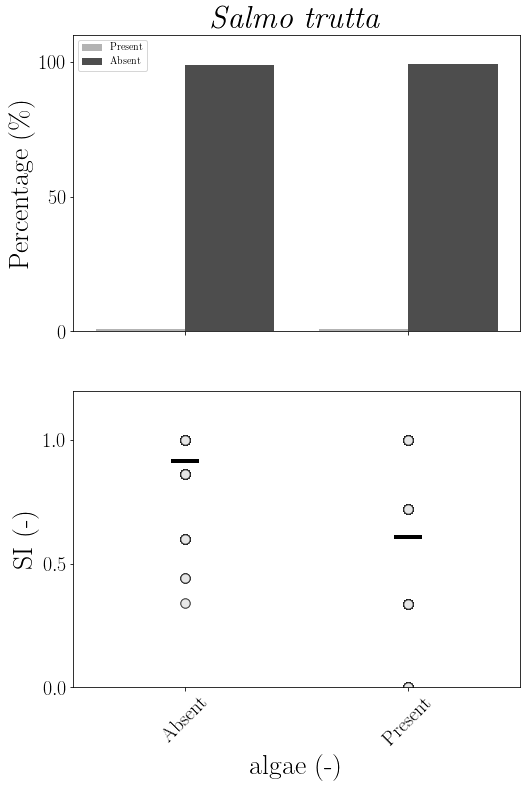
**
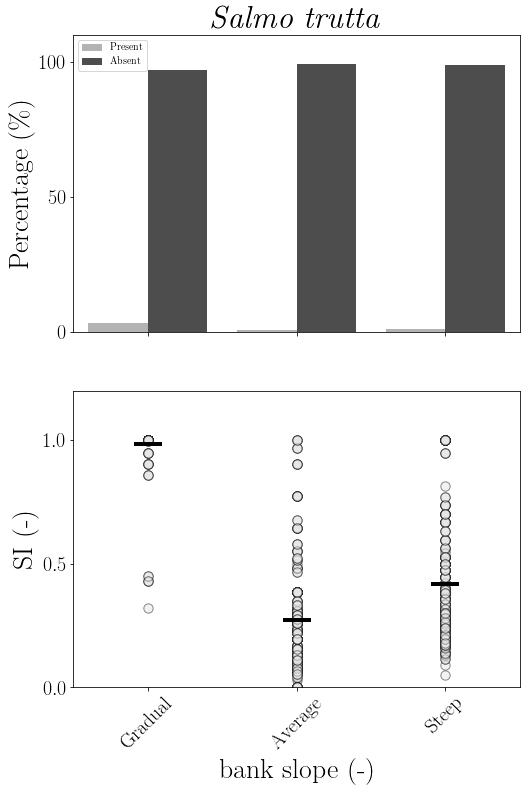

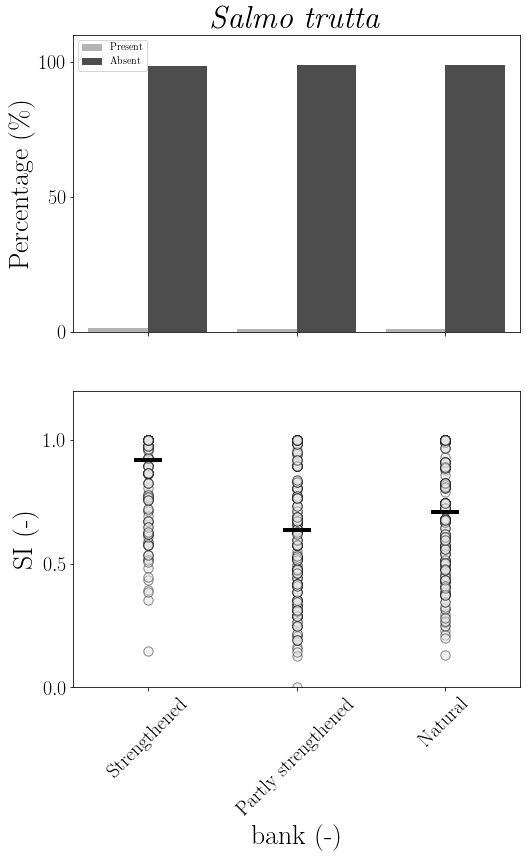

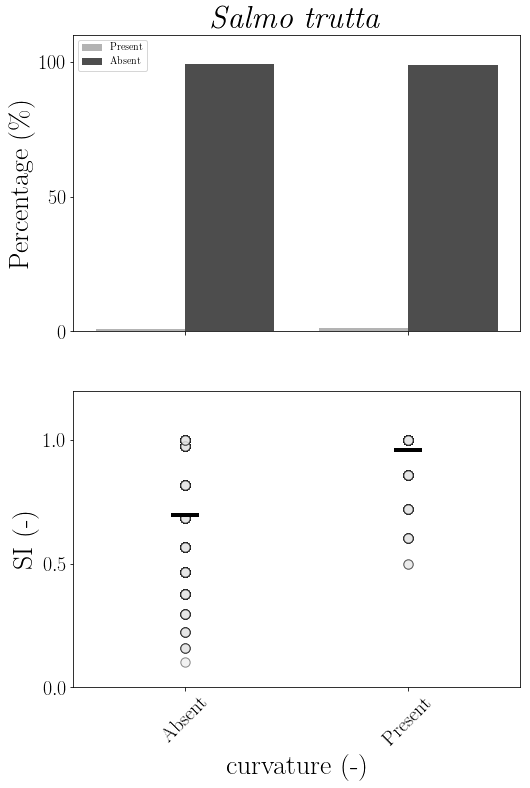


**Figure D1: Presence-absence for every class and estimated SI for categorical and binary variables (algae, bank slope, bank, curvature). Every individual points presents one bootstrap and the median is indicated with the black line.**


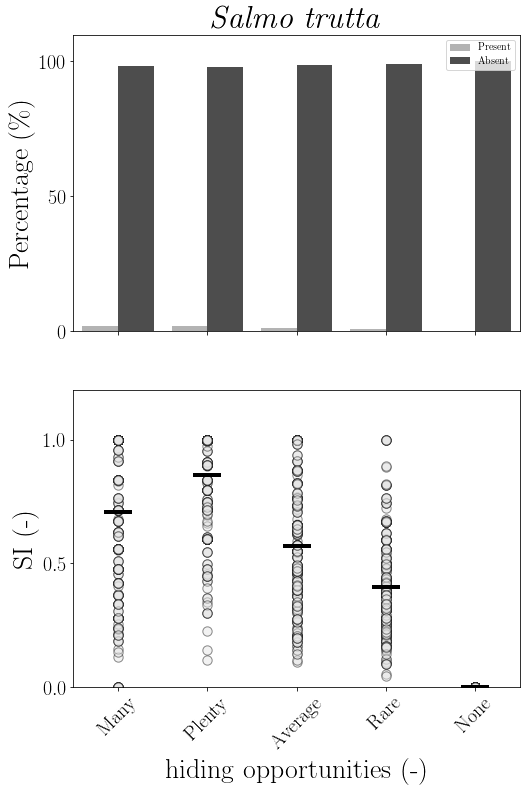

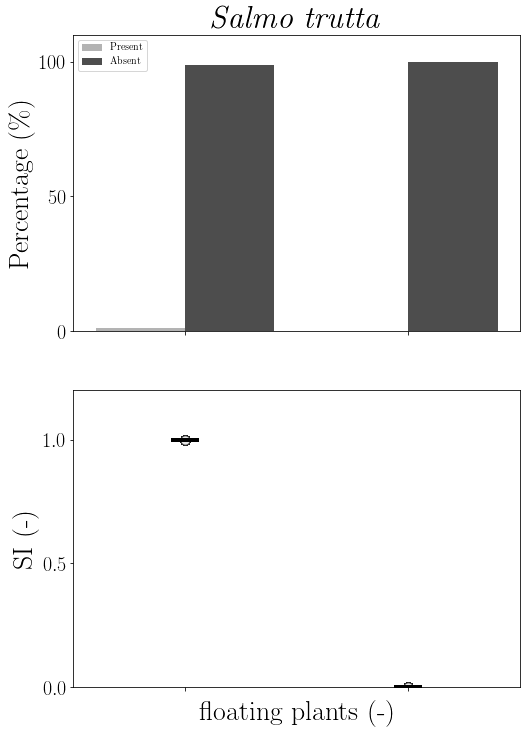

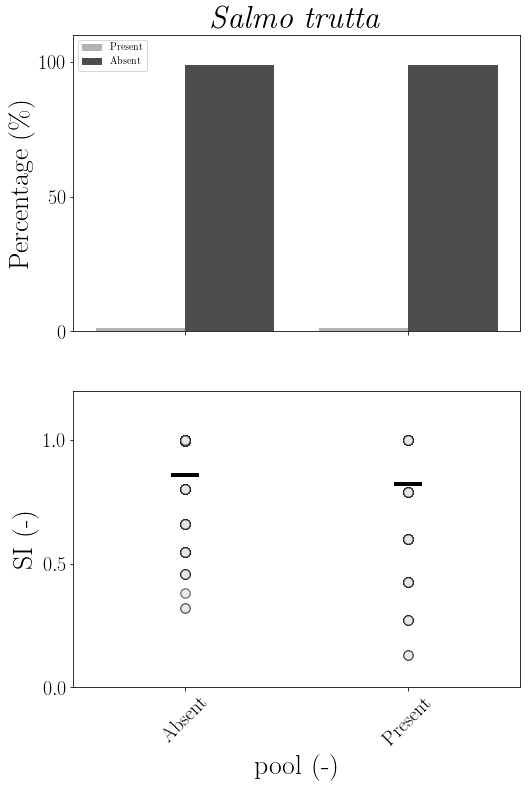

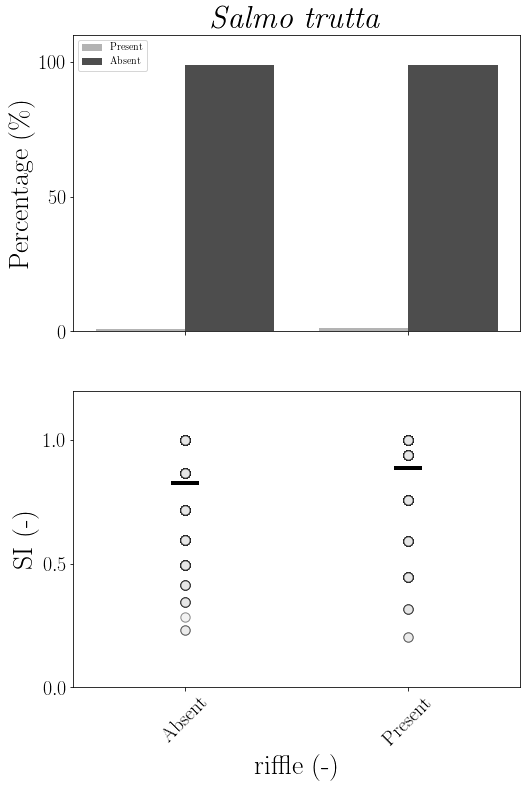


**Figure D2: Presence-absence for every class and estimated SI for categorical and binary variables (hiding opportunities, non submerged plants, pool and riffle). Every individual points presents one bootstrap and the median is indicated with the black line.**

**
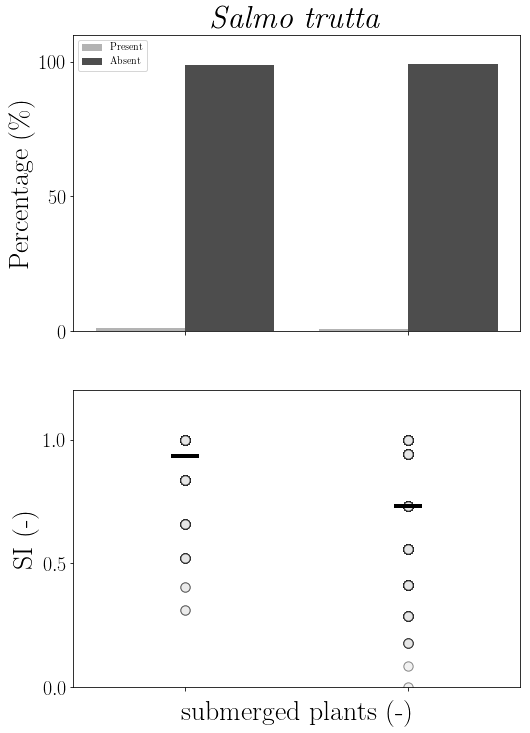

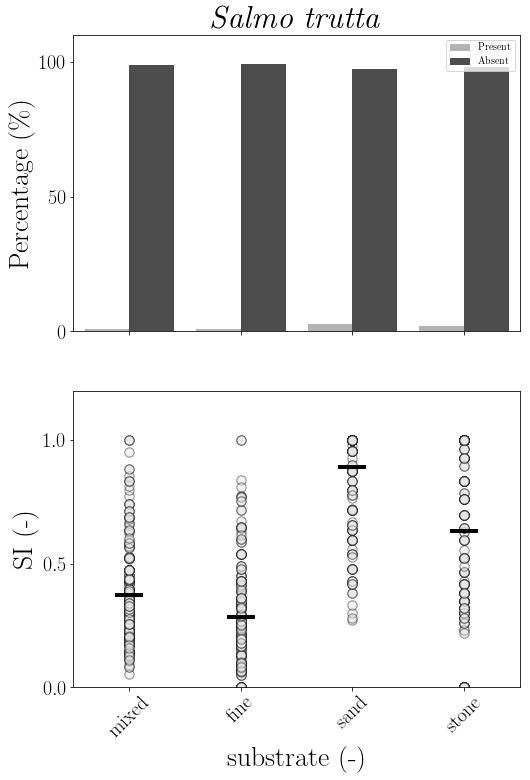
**

**Figure D3: Presence-absence for every class and estimated SI for categorical and binary variables (submerged plants and substrate). Every individual points presents one bootstrap and the median is indicated with the black line.**


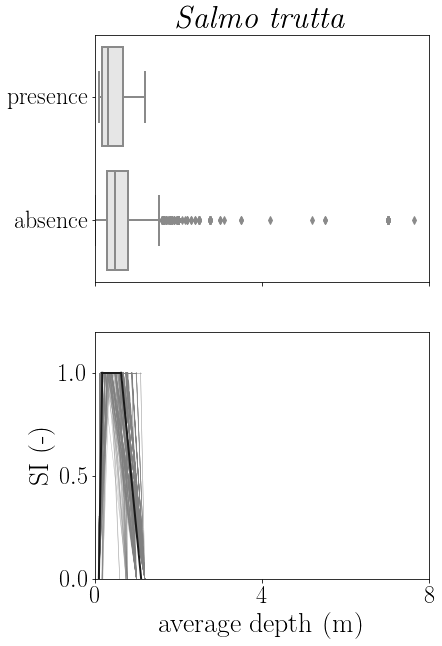

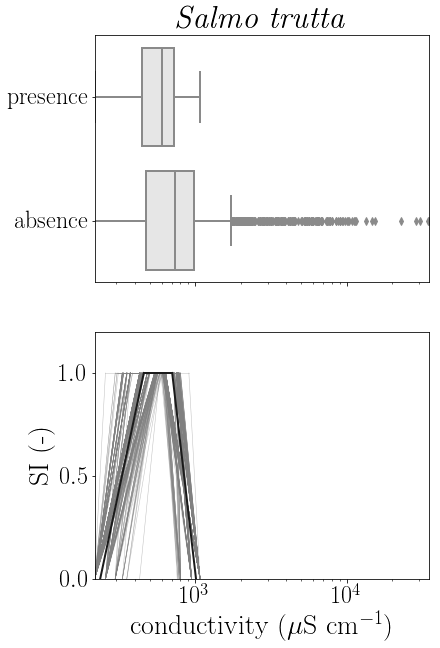

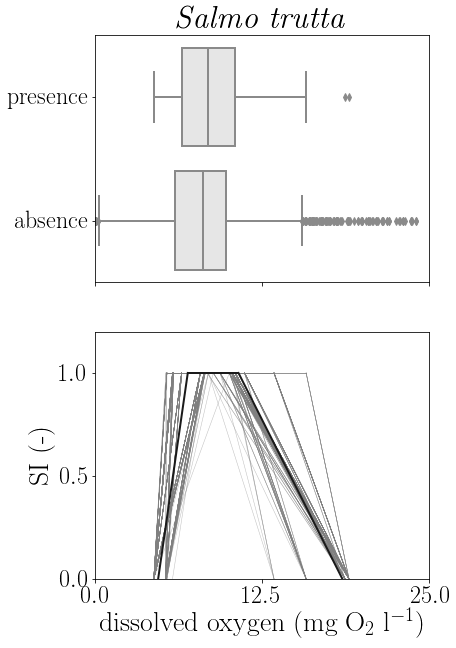

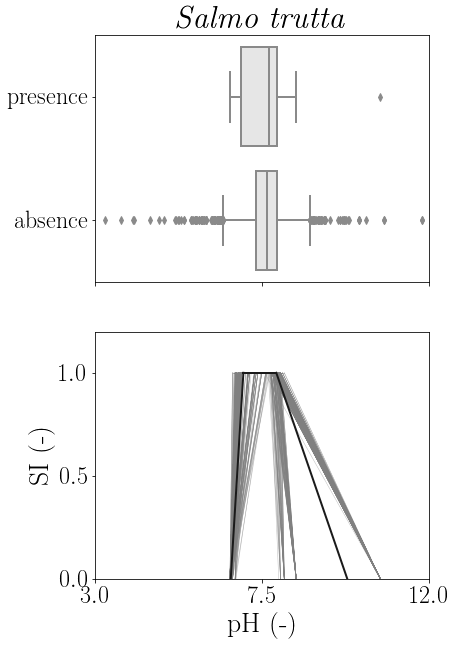


**Figure D4: Boxplot of values for presence and absence of the species and estimated SI for continuous variables (average depth, conductivity, dissolved oxygen and pH). Every individual curve presents one bootstrap and the curve constructed from the median values of a_1_, a_2_, a_3_ and a_4_ is indicated in black.**


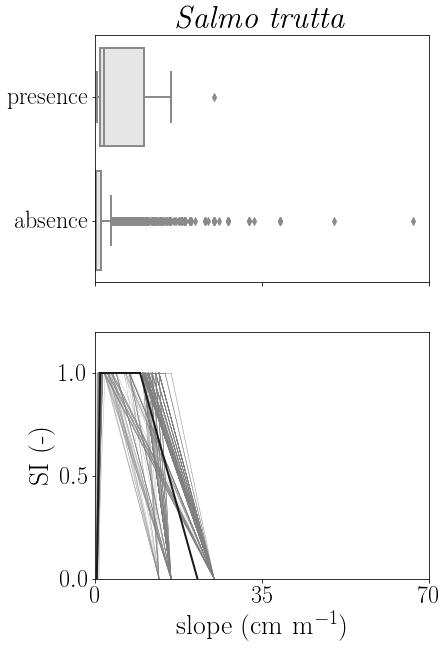

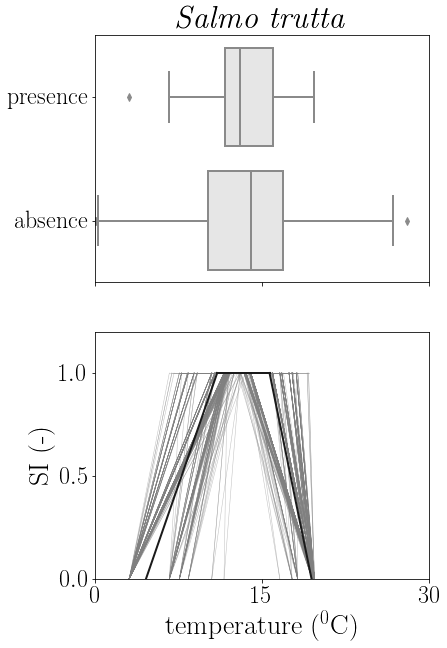
**
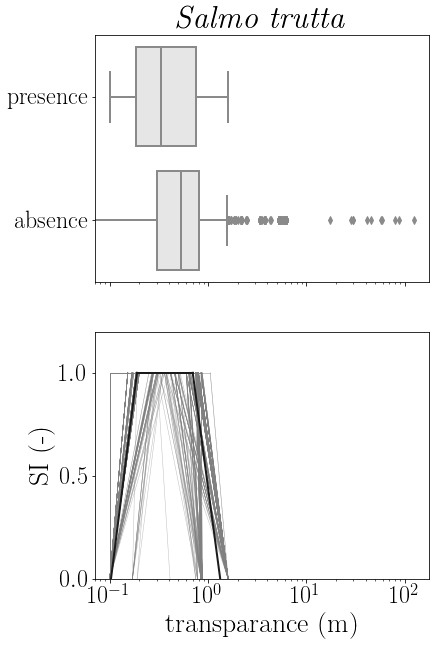

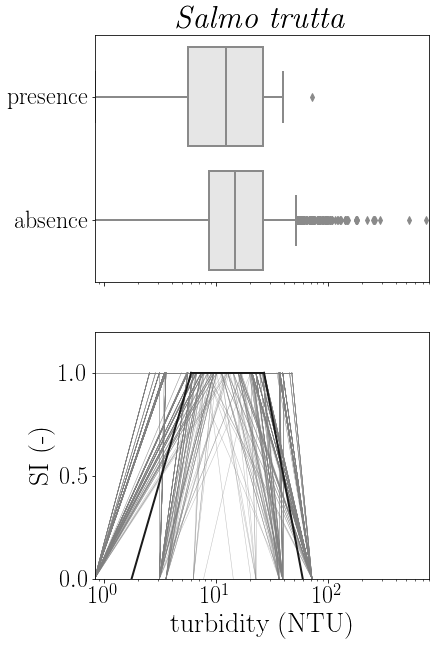
**

**Figure D5: Boxplot of values for presence and absence of the species and estimated SI for continuous variables (slope, temperature, transparency and turbidity). Every individual curve presents one bootstrap and the curve constructed from the median values of a_1_, a_2_, a_3_ and a_4_ is indicated in black.**

**
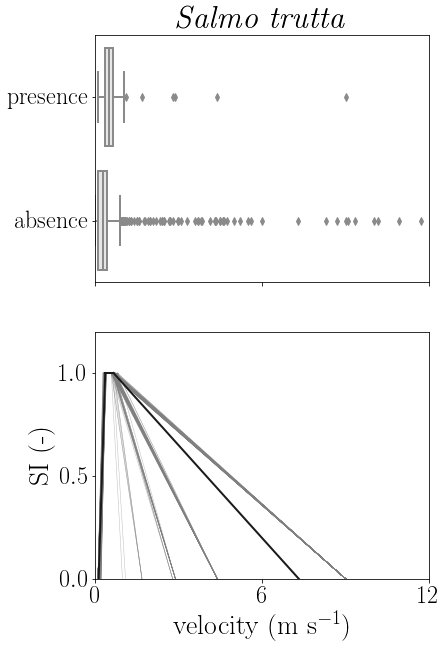

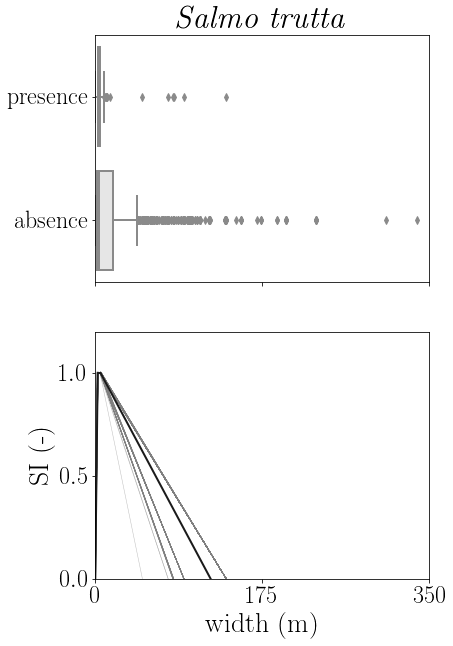
**

**Figure D6: Boxplot of values for presence and absence of the species and estimated SI for continuous variables (velocity and width). Every individual curve presents one bootstrap and the curve constructed from the median values of a_1_, a_2_, a_3_ and a_4_ is indicated in black.**

**References**

Araújo, M.B. & New, M. (2007) Ensemble forecasting of species distributions. Trends in Ecology & Evolution, **22**(1), 42-47.

Ellison, A.M. (2004) Bayesian inference in ecology. Ecological Letters, **7**, 509–520.

Gibbs, M.S., Dandy, G.C. & Maier, H.R. (2008) A genetic algorithm calibration method based on convergence due to genetic drift. Information Sciences, **178**, 2857–2869.

Gibbs, M.S., Maier, H.R. & Dandy, G.C. (2010) Comparison of genetic algorithm parameter setting methods for chlorine injection optimization. Journal of Water Resources Planning and Management, **136**, 288–291.

Gobeyn, S., Martin. V., Dominguez-Granda, L. & Goethals, P.L.M. (2017) Input variable selection with a simple genetic algorithm for conceptual species distribution models: A case study of river pollution in Ecuador. Environmental Modelling and Software, **92**, 269–316.

Goldberg, D.E. (1989) Genetic Algorithms in Search, Optimization, and Machine Learning. Addison-Wesley Longman Publishing Co., Inc., Boston.

Maier, H.R., Kapelan, Z., Kasprzyk, J., Kollat, J., Matott, L.S., Cunha, M.C., Dandy, G.C., Gibbs, M.S., Keedwell, E., Marchi, A., Ostfeld, A., Savic, D., Solomatine, D.P., Vrugt, J.A., Zecchin, A.C., Minsker, B.S., Barbour, E.J., Kuczera, G., Pasha, F., Castelletti, A., Giuliani, M. & Reed, P.M. (2014) Evolutionary algorithms and other metaheuristics in water resources: Current status, research challenges and future directions. Environmental Modelling and Software, **62**, 271–299.

Shannon, C.E. (1948) The mathematical theory of communication. The Bell System Technical Journal, **27**, 379-423.
